# Domain-specific and multidomain resilience among parentally bereaved youth: Assessment and associations with long-term outcomes

**DOI:** 10.1017/S0954579425101107

**Published:** 2026-02-10

**Authors:** Irwin Sandler, Qiyue Cai, Jenn-Yun Tein, Rebecca Hoppe, Sharlene Wolchik

**Affiliations:** Department of Psychology, Arizona State University, Tempe, AZ, USA

**Keywords:** Bereaved children, domain-specific resilience, growth mixture modeling, multidomain resilience

## Abstract

This study addressed the prevalence of resilience within specific domains (domain-specific resilience) and across multiple domains (multidomain resilience), as well as the predictive value of resilience for long-term outcomes. Using data from 244 parentally bereaved youth ages 8 – 16 who completed multiple assessments over 15 years in a randomized preventive intervention trial, we examined resilience trajectories across 10 outcomes in five domains on which bereaved youth are at risk, assessed over 14 months. Resilience was defined as low, stable problems or high, stable competencies across assessments; and multidomain resilience as the number of outcomes on which there were resilient trajectories. Results showed that resilience was generally common within specific domains, though its prevalence varied across multiple domains. Multidomain resilience followed a near-normal distribution, with few people having no domain on which they are resilient or being resilient across all domains. Several domain-specific resilience trajectories and multidomain resilience predicted multiple outcomes 15 years after baseline.

## Introduction

The construct of resilience involves two key components: experiencing adversity and demonstrating positive or better-than-expected adaptation following exposure to adversity (Luthar et al., 2000; Luthar, 2015; Rutter, 2012). Adversity refers to negative life events that are empirically found to increase risk for later adaptation problems. Positive adaptation has been assessed in resilience research using indicators of competent functioning (e.g., success in age-appropriate role performance) and low levels of mental health, physical health, or behavioral problems. Consistent with the principle of multifinality (Cicchetti & Rogosh, 1996), children who are exposed to major adversities such as bereavement (Dowdney, 2000), divorce (Amato, 2014), and abuse (Xiao et al., 2023) are at increased risk for multiple adjustment problems. How to account for the multiple adjustment problems following adversity poses an important challenge for resilience research. In this paper, we present an approach to assessing positive adaptation over time among parentally bereaved youth using indicators of competence and adjustment in five domains of functioning, which prior research has found to be associated with the adversity of the death of a parent during childhood.

An important methodological advance in resilience research over the past two decades has been the use of a person-focused approach, such as growth mixture modeling (GMM; Muthén et al., 2002), to group individuals with similar trajectories of adapting to adversities over time (Infurna & Grimm, 2018). GMM or an alternative, latent class growth analysis (LCGA; Nagin, 2005), is a statistical technique used to identify unobserved, distinct trajectories of individuals’ outcomes based on similar patterns of responses over multiple time points. This approach differs from a variable-focused approach of growth curve modeling which examines the trajectory of change on a variable over time, assuming that all individuals are drawn from a single population with common population parameters (i.e., similar baseline scores and growth trajectories). GMM allows for differences of growth parameters across unobserved subpopulations (Muthén, 2004). By using GMM, we can assess trajectories of competence and adjustment in multiple domains of functioning for bereaved youth following the death of their parent.

A meta-analysis of 54 GMM studies of children and adults who had experienced a range of traumatic events (Galatzer-Levy et al., 2018) identified four distinct trajectories of common mental health problems over time. The most common trajectory, resilience, was characterized by stable low levels of mental health problems, with a pooled prevalence rate of 65.7% across studies. Studies of children within this same review (*n* = 6 studies) also found that resilience was the most common trajectory across multiple single outcomes, with a 52% pooled prevalence rate. Based on these findings, the authors concluded that resilience is a common and modal trajectory following a wide range of adversities.

One caveat to this conclusion is that the review only reported on the single “primary outcomes” and did not include reports on resilient trajectories on multiple domains of problems or developmental competence. Prior research has argued that children’s resilience following adversity needs to account for variability in adjustment across different domains of functioning, including positive developmental outcomes (Luthar & Eisenberg, 2017). Luthar et al. (2000) noted that heterogeneity across domains of functioning is to be expected and found that children who exhibit successful functioning in one domain often exhibit problems in other domains. Illustratively, Luthar et al. (1993) in a sample of inner-city adolescents found that resilience in academic functioning was not matched by resilience in emotional well-being. As children are at risk for problems in multiple domains of functioning following adversity, Luthar and colleagues (2000) proposed that it makes sense to assess resilience in each domain separately. We follow this approach in the current study of resilience across domains of functioning for parentally bereaved youth. Key research questions concern the prevalence of resilience for each at-risk domain as well as across domains, and the extent to which domain-specific and multidomain resilience predict later adjustment.

Prior research with adult populations has demonstrated that while resilience is often the modal trajectory for single outcomes following adversity, its prevalence varies by outcome, and resilience across multiple outcomes is rare (referred to as multidimensional resilience, Infurna & Luthar, 2017a; 2017b). In a series of studies of resilience for bereaved adults (Infurna & Luthar, 2017a, 2017b), the authors found differential rates of resilience across multiple domains of physical and mental health functioning. Specifically, the percentage of people who showed a resilient trajectory to spousal loss varied across five domains: life satisfaction (66%), negative affect (19%), positive affect (26%), general health (37%), and physical functioning (28%). Very few people (8%) had resilient trajectories across all five domains, and 20% did not show a single resilient trajectory in any of the domains (Infurna & Luthar, 2017a). Similar findings were observed for parents who had experienced child loss (Infurna & Luthar, 2017b). Although they report high prevalence of resilience in some domains, they concluded that resilience across multiple outcome domains is rare.

The current study assesses trajectories of resilience across multiple domains of problems and competencies on which they are at risk of problem outcomes. We use both self- and caregiver-reports of outcomes where available in a sample of parentally bereaved youth. This is only the third study to assess trajectory patterns of outcomes for parentally bereaved youth. In a sample of youth who experienced the sudden death of a parent, Melhem et al. (2011) identified three distinct trajectories of children’s grief over 33 months: a modal trajectory of low levels of grief that decreased over time (58.8%), moderate levels of grief that decreased over time (30.8%), and high levels of grief that remained high over time (10.4%). Using a sample of 244 parentally bereaved youth, we partially replicated these findings on trajectories of grief and extended them over six years (Sandler et al., 2025).

In the current study, we use this sample to extend research on resilience of parentally bereaved youth by assessing trajectories of 10 outcomes in five domains for which parentally bereaved youth had previously been found to be at elevated risk, including higher levels of prolonged intrusive grief (Sandler et al. 2025; Kaplow et al., 2012; Melhem et al., 2011), internalizing problems (Berg et al., 2016; Böckerman et al., 2023; McKay et al., 2022; Simbi et al., 2020), and externalizing problems (Hamdan et al., 2012; Kaplow et al., 2010; Melhem et al., 2011; Luecken & Rubinov, 2012), as well as lower levels of academic competence (Brent et al., 2012; Liu et al., 2022; Oosterhoff et al., 2018), and peer competence (Brent et al., 2012). We study the prevalence of domain-specific resilience for each of these outcomes and multidomain resilience as the cumulative number of domains on which youth had resilient trajectories. Similar to research with adults (Infurna & Luthar, 2017a, 2017b), we expected that resilience may be the modal trajectory for individual outcomes, but very few people would be considered resilient across all domains or have no domain being considered as resilient. Finally, we test the long-term predictive value of both domain-specific and multidomain resilience for outcomes assessed 15 years after baseline.

## Method

### Participants

Participants included 244 children and adolescents (hereafter referred to as youth) and their caregivers from 156 families who participated in a randomized controlled trial of the Family Bereavement Program (FBP). Families had at least one youth between 8 and 16 years of age (*M* = 11.29, *SD* = 2.43, 54% boys) who experienced the death of a parent between 3 and 30 months prior to entering the trial (*M* = 10.81, *SD* = 6.35) and were fluent in English. Youth identified their race/ethnicity as follows: non-Hispanic Caucasian (67%), Hispanic (16%), African American (7%), Native American (3%), Asian or Pacific Island (1%), and Others (6%). The reported causes of death included illness (67%), accident (20%), and violence (suicide or homicide; 13%).

### Procedure

All study procedures were approved by the university’s Institutional Review Board. Full details on eligibility, recruitment, and study procedure were described elsewhere (Sandler et al., 2003; Sandler et al., 2010; Sandler et al., 2018). Briefly, families were recruited between 1996 and 1999 through local community agencies that had contact with bereaved youth and families (e.g., schools, churches, hospices), newspapers, and media presentations. After the baseline assessment, eligible families were randomized into either the FBP intervention condition (*n* = 135 youth from 90 families) or the self-study condition (*n* = 109 youth from 66 families). Families were interviewed and assessed on all measures used in the current study at baseline (W1) and at three months (W2; 98% retention), 14 months (W3; 90% retention), and 15-years (77% retention) post baseline. The 15-year outcomes were assessed between 2011 to 2014. Families and youth received compensation for participating in each assessment. All participants provided informed consent or assent at each assessment.

### Measures

Ten measures were administered from W1 – W3 for our assessment of GMM trajectories. Measures were classified within five domains of functioning, including grief, internalizing problems, externalizing problems, academic competence, and peer competence. For all domains except grief, measures were administered to both youth and caregivers, allowing us to classify youth as resilient on each domain using the trajectories showing agreement across both reporters and by either reporter. We reported internal consistency (Cronbach’s α) of each scale at baseline. The internal consistency scores of the later waves were similar (i.e., different by the second decimal place) and thus are not reported here.

#### Domain 1: Grief

##### Intrusive grief, youth-report

Youth intrusive grief was measured by the Intrusive Grief Thoughts Scale developed for the FBP study (Sandler et al., 2010; *α* = .89) at W1 – W3. Youth rated 10 statements reflecting the frequency of experiencing intrusive and unwanted negative or impairing thoughts regarding the death of their parent on a 5-point Likert scale from 1 (*not at all*) to 5 (*several times a day*). Higher mean scores indicated more intrusive grief symptoms.

#### Domain 2: Internalizing problems

##### Depression, youth-report

Youth depression was measured by the Children’s Depression Inventory (CDI; Kovacs, 1981; *α* = .87). Youth rated 27 statements from 0 (*absence of symptoms*) to 2 (*definite symptom*) in the past two weeks. Higher sum scores indicated more severe depressive symptoms.

##### Anxiety, youth-report

Youth anxiety was measured by the Revised Children’s Manifest Anxiety Scale (RCMAS; Reynolds & Richmond, 1978; *α* = .90). Youth responded with 1 (*no*) or 2 (*yes*) to 28 statements reflecting anxiety symptoms. Higher sum scores indicated more severe anxiety symptoms.

##### Internalizing problems, caregiver-report

Caregivers reported children’s internalizing problems using the Child Behavioral Checklist (CBCL; Achenbach, 1991a) for youth under 18 years old. Age- and sex- adjusted *T*-scores were calculated (*M* = 50, SD = 10).

#### Domain 3: Externalizing problems

##### Externalizing problems, youth-report

Youth reported their own externalizing problems using the Youth Self Report (YSR; Achenbach, 1991b). Age- and sex- adjusted T-scores were calculated (*M* = 50, SD = 10).

##### Externalizing problems, caregiver-report

Caregivers reported children’s externalizing problems using the Child Behavioral Checklist (CBCL; Achenbach, 1991a) for youth under 18 years. Age- and sex- adjusted *T*-scores were calculated (*M* = 50, SD = 10).

#### Domain 4: Academic competence

##### Academic competence, youth- and caregiver-report

Youth self-report and caregiver-report of academic competence were measured by the subscale from the Coatsworth Competency Scale (Coatsworth & Sandler, 1993). Youth and caregivers rated six parallel statements about the youth’s academic competence on a 4-point Likert Scale from 1 (*not at all*) to 4 (*very much*). This subscale showed good internal consistency in the current sample (*α* = .79 for youth-report, *α* = .89 for caregiver-report).

#### Domain 5: Peer competence

##### Peer competence, youth- and caregiver-report

Similar to academic competence, youth self-report and caregiver-report of peer competencies were measured by the 7-item subscale from the Coatsworth Competency Scale (Coatsworth & Sandler, 1993). The subscale showed acceptable internal consistency in the current sample (*α* = .64 for youth-report, *α* = .78 for caregiver-report). While the youth-report showed lower-than-ideal internal consistency, it was moderately associated with caregiver report (*r* = .27) and youth reports of anxiety and depression (both *r* = .30).

#### Outcomes at 15 Years for analysis of long-term prediction by domain-specific and multidomain resilience

##### Intrusive grief thoughts, youth-report

Intrusive grief was measured by the same 10-item Intrusive Grief Thoughts Scale (*α* = .90) developed for the FBP study (Sandler et al., 2010).

##### Social detachment/Insecurity, youth-report

Social detachment/insecurity was measured by the 7-item subscale derived as a specific grief dimension that was not correlated with general grief based on a bi-factor analysis of the Inventory of Traumatic Grief (Prigerson & Jacobs, 2001; Sandler et al., 2010; *α* = .85). Youth rated each item on a 5-point Likert scale, and higher scores indicated more grief-related social detachment and insecurity.

##### Personal growth through grief, youth-report

Youth personal growth through grief was measured using the 12-item personal growth subscale from the Hogan Grief Reaction Checklist (HGRC; Hogan et al., 2001; *α* = .94). The scale assessed youth’s sense of having become more compassionate, tolerant, forgiving, and hopeful post-bereavement on a 5-point Likert scale from 1 (*does not describe me at all*) to 5 (*describes me very well*). Higher scores indicate more personal growth.

##### Internalizing problems, youth-report

Internalizing problems during the past month were assessed using the Adult Self-Report (ASR; Achenbach & Rescorla, 2003; *α* = .93).

##### Major depressive disorder (MDD)

The binary MDD diagnosis variable was assessed by the World Health Organization World Mental Health Composite International Diagnostic Interview (CIDI; Robins et al., 1988). Using computerized algorithms, the CIDI defines whether the individual meets all criteria for major depressive disorder as defined by the DSM-IV and ICD-10 (0 = no MDD diagnosis, 1 = MDD diagnosis).

##### Externalizing problems, youth-report

Externalizing problems during the past month were assessed using the Adult Self-Report (ASR; Achenbach & Rescorla, 2003; *α* = .90).

##### Polysubstance use, youth-report

Youth polysubstance use was measured by the sum scores of Tobacco, Alcohol, and Drug use subscales from the Adult Self-Report (ASR; Achenbach & Rescorla, 2003). Internal consistency was not applicable for this measure.

##### Suicidality, youth and caregiver report

Suicidal risk was assessed using a binary variable (0 = *no suicidal risk*, 1 = *experiencing suicidal ideation or attempts*) by two items from YASR (Achenbach, 1997) reported by youth and key informants (nominated by the youth). Youth reported on the items that happened in the past month (i.e., “Deliberately harms self or attempts suicide” and “Talks about killing self”) while the key informants reported on the parallel items that happened in the last six months (see Sandler et al., 2016). Suicidal ideation or attempts were considered present if endorsed by either the youth or key informants.

##### Mastery, youth-report

Mastery was assessed by a modified 10-item Mastery scale (Pearlin & Schooler, 1978; *α* = .86). Youth rated items on a 4-point Likert scale from 1 (*strongly disagree*) to 4 (*strongly agree*). Higher scores indicate higher mastery.

##### General health, youth-report

Youth rated one item from SF-12 (Ware et al., 1996), “how would you describe your general health” from 1 (*Poor*) to 5 (*Excellent*).

#### Demographic variables

Youth age at parental death (in years), sex (0 = *boy*, 1 = *girl*), cause of parental death (two dummy-coded variables for 1 = *accident* vs. 0 = *illness*; 1 = *violent* vs. 0 = *illness*), and intervention status (0 = *control*, 1 = *FBP*) were assessed at baseline.

### Analytic plan

We conducted analyses in four phases: (1) examined trajectory patterns across W1 – W3 for each of the grief, mental health, and competency variables using GMM, (2) defined the resilience group for each of the 10 variables as *better than expected* across each of the three assessments in the trajectory using national norms or scores for bereaved children (Luthar, 2015; Rutter, 2012); identified resilience for each of the five domains (i.e., domain-specific resilience) based on agreement across reporters and reports by either reporter; and calculated multidomain resilience scores as the number of domains on which youth were considered resilient based on agreement across trajectories of both reporters or resilient trajectory assessed by either reporter, (3) examined the relationship between multidomain resilience scores and baseline demographics as well as intervention status using Poisson regressions, and (4) examined the associations between grief, mental health, mastery and general health outcomes at 15-years post-baseline and the prevalence of domain-specific and multidomain resilience using linear and logistic regressions as appropriate. Phases 1, 3, and 4 were conducted in Mplus (Muthén & Muthén, 1998-2017).

#### Phase 1: Growth trajectory patterns

For each variable, we fit a series of GMMs to identify trajectory patterns over time following the date of parental death and estimate the posterior probability of each individual being a member of each profile. Instead of using the conventional fixed time for the assessments (i.e., W1 – W3), the time since death was the time indicator. In the current study, youth experienced parental death between 3 and 30 months prior to baseline. As a result, the timeframe for all participants ranged from 3 to 44 months. For example, youth who lost a parent three months before baseline were assessed at approximately 3, 6, and 17 months since death whereas those whose loss occurred 30 months before the baseline were assessed at 30, 33, and 44 months since death. For ease of modeling, we compressed the time unit to every 4 months. Mplus settings for conducting GMMs leverage all available data. Thus, data from youth having at least one assessment on the variable were included in the growth models. However, the exact time of death for four youth was missing, and those cases were excluded from the GMM. We accounted for family clustering by computing robust standard errors using a sandwich estimator (Muthén, 1998).

GMM is an exploratory data analysis; we started with a 1-profile model and successively increased the number of profiles by one until model fit indices leveled off or we encountered convergence issues (Ram & Grimm, 2009). We tested both linear and quadratic growth models. When the two growth models were comparable in fit, we selected the quadratic model only if one or more quadratic terms within the latent classes were statistically significant. To avoid getting local maximum solutions, we repeated models with multiple sets of start values and ensured that the best log-likelihood value was replicated (Muthén, 2004). There is no single fit index that is best for optimal class solution for the mixture modeling (Nylund-Gibson & Choi, 2018; Tein et al., 2013). We thus determined the optimal number of profiles based on several fit indices and likelihood ratio tests: Bayesian information criterion (BIC; Schwarz, 1978), sample-size adjusted Bayesian information criterion (saBIC; Sclove, 1987), and Vuong-Lo-Mendell-Rubin likelihood ratio test (LMR; Lo et al., 2001). We could not perform a parameter bootstrapped likelihood ratio test (Peel & McLachlan, 2000) due to the clustering effect of siblings. We also relied on entropy to gauge whether the latent profiles were highly discriminating (Nylund et al., 2007; Ram & Grimm, 2009) and substantive interpretations for model selection criteria. Good entropy is generally defined as > .80, with lower entropy being acceptable (e.g., > .70) if the other fit indices favor the solution; but there is no agreed-upon cutoff point for poor entropy (Muthén, 2017). Similarly, for the LMR test, a *p*-value ≤ .05 is preferred to indicate that the K_0_-class solution is significantly better than the K_-1_- class solution. However, in case LMR did not agree with other fit indices, we rely on information criterion (BIC, saBIC) and interpretability (Muthén, 2019) to identify the optimal solution. Each youth was assigned to the most likely profile based on the estimated posterior probabilities for each profile for the computation of multidomain resilience scores in the next phase.

#### Phase 2: Prevalence of domain-specific and multidomain resilience

After identifying the best-fitting model for each variable, we defined the resilience groups for each of the 10 outcome variables across the five domains. We defined resilience based on the widely used conceptualization of resilience as stable low problems or stable high competence (2016b; Bonanno & Diminich, 2013; Galatzer-Levy et al., 2018; Galatzer-Levy & Bonanno, 2016, Infurna & Luthar, 2016a). While the classification of “stable low” or “stable high” trajectories is somewhat subjective, we adopt Rutter’s (2012) notion of “better-than-expected” outcomes for our operationalized definition. Consistent with Melhem et al. (2011), “expected” outcomes were operationalized as the baseline sample mean, with additional reference to subclinical (when available) or clinical cutoffs and the substantive meaning of the rating scale when applicable. Thus, we categorized a group as resilient if the estimated trajectory remained entirely below the baseline mean for symptom measures or above the baseline mean for competence measures, with additional reference when applicable. Specifically, for the variables with clinical cutoff scores (i.e., CDI and RCMAS) or standardized norms (i.e., CBCL and YSR *T*-scores), the entire estimated trajectory for the resilience group was required to remain below the (marginal) clinical cutoff scores. For those without clinical cutoff scores or national norms (grief and competence scales), substantive meaning of the rating scales and a score above the mean of academic and social competence and below the mean on grief in this sample was used to satisfy the resilience definition. For each variable, youth received a score of 1 for being in the resilience group or 0 for non-resilience. This definition emphasized that resilience is not a static construct assessed at a single time point but reflects the *stability* of positive or better-than-expected outcomes over time.

Four of the five domains (excluding grief) included both youth- and caregiver-report measurement. As there had been long-term debate on who served as better reporter of child behaviors (e.g., Achenbach et al., 1987), we calculated two types of domain-specific resilience: (a) resilience based on agreement across reporters (1 = agreement across youth and caregiver reports of resilience; 0 = at least one report of non-resilience), and (b) resilience by either reporter (1 = at least one report from either youth or caregiver indicated resilience; 0 = non-resilience by the other reporter). For example, youth were classified as resilient in the externalizing problems domain across reporters when both youth and caregiver reports indicated resilience, and as resilient by either reporter when resilience was indicated by at least one of the reporters. Youth were considered resilient on internalizing problems across reporters when they received a score of 1 on all three measures: youth-report anxiety, youth-report depression, and caregiver-report of internalizing problems. Youth were considered resilient on internalizing problems by either reporter if they received a score of 1 on either of the three measures. Similarly, two types of multidomain resilience scores were calculated by summing the resilience across the five domains for (a) agreement across reporters and (b) either-reporter definitions (range = 0 – 5). (Table 1–4)

**Table 1.** Summary of growth mixture models analyses

Outcome	Reporter	Final class solution	Modal trajectory	Resilience single outcome	Resilience across reporters	Resilience either reporter
***Grief***					87 (36.3%)	87 (36.3%)
Intrusive Grief	Youth	3-class linear	No	87 (36.3%)		
***Internalizing Problems***					107 (44.6%)	219 (91.3%)
Depression	Youth	3-class quadratic	Yes	198 (82.5%)		
Anxiety	Youth	3-class quadratic	Yes	154 (64.2%)		
Internalizing Problems	CG	4-class quadratic	Yes	149 (62.1%)		
***Externalizing Problems***					95 (39.6%)	191 (79.6%)
Externalizing Problems	Youth	2-class quadratic	Yes	149 (62.1%)		
Externalizing Problems	CG	5-class linear	Yes	137 (57.1%)		
***Academic Competence***					113 (47.1%)	189 (78.8%)
Academic Competence	Youth	2-class linear	Yes	138 (57.5%)		
Academic Competence	CG	2-class linear	Yes	164 (68.3%)		
***Peer Competence***					116 (48.3%)	203 (84.6%)
Peer Competence	Youth	2-class linear	Yes	182 (75.8%)		
Peer Competence	CG	3-class quadratic	Yes	137 (57.1%)		

*Note.* Youth = youth self-report, CG = caregiver report.

**Table 2.** Frequency distribution of multidomain resilience indexes

Resilience across reporters			Resilience either reporter		
# of resilient classes	*n*	%	# of resilient classes	*n*	%
0	31	12.9%	0	2	0.8%
1	60	25.0%	1	13	5.4%
2	55	22.9%	2	14	5.8%
3	45	18.8%	3	57	23.8%
4	32	13.3%	4	93	38.8%
5	17	7.1%	5	61	25.4%

**Table 3.** Poisson regression analyses of demographic variables on multidimensional resilience indexes

	Resilience across reporters		Resilience either reporter	
	b (SE)	*P*_*FDR*_	b (SE)	*P*_*FDR*_
Youth Age	0.02 (0.01)	.120	0.002 (0.003)	.782
Youth Sex	0.01 (0.09)	.887	−0.002 (0.04)	.962
Accident	−0.31 (0.15)	.120	−0.10 (0.06)	.555
Violence	0.08 (0.17)	.810	−0.03 (0.08)	.856
Intervention	0.08 (0.09)	.705	0.03 (0.04)	.782

*Note. P*_*FDR*_ = False Discovery Rate (FDR) adjusted *p*-value.

*p < .05, ** *p* < .01, *** *p* < .001.

**Table 4.** Regression analyses of mental health, grief, health and mastery 15-years post baseline on domain-specific and multidomain resilience

	Domain 1 grief		Domain 2 internalizing problems		Domain 3 externalizing problems		Domain 4 academic competence		Domain 5 peer competence		Multidomain resilience	
	*b* (*SE*)	*P*_*FDR*_	*b* (*SE*)	*P*_*FDR*_	*b* (*SE*)	*P*_*FDR*_	*b* (*SE*)	*P*_*FDR*_	*b* (*SE*)	*P*_*FDR*_	*b* (*SE*)	*P*_*FDR*_
**Resilience across Reporters**												
Intrusive Grief	**−0.36 (0.07)**	**< .001**	**−0.22 (0.08)**	**.014**	−0.14 (0.09)	.201	**−0.26 (0.09)**	**.014**	−0.12 (0.09)	.314	**−0.13 (0.03)**	**< .001**
Grief-related Social Detachment/Insecurity	−0.13 (0.09)	.221	**−0.38 (0.08)**	**< .001**	**−0.24 (0.09)**	**.035**	−0.17 (0.09)	.157	−0.19 (0.09)	.099	**−0.14 (0.03)**	**< .001**
Personal Growth Through Grief	**0.48 (0.12)**	**< .001**	0.19 (0.13)	.228	0.29 (0.13)	.055	0.22 (0.14)	.221	−0.08 (0.14)	.654	**0.13 (0.04)**	**.012**
Internalizing Problems	−0.88 (1.94)	.698	**−6.44 (1.53)**	**< .001**	−1.36 (1.83)	.562	**−4.07 (1.72)**	**.047**	−2.33 (1.85)	.304	**−1.86 (0.59)**	**.009**
MDD Diagnosis	−0.35 (0.51)	.595	−0.26 (0.42)	.620	−0.31 (0.38)	.536	−0.28 (0.44)	.620	−0.52 (0.45)	.347	−0.21 (0.15)	.261
Externalizing Problems	−2.36 (1.54)	.221	**−4.43 (1.35)**	**.005**	**−3.72 (1.49)**	**.036**	**−3.83 (1.51)**	**.035**	0.84 (1.57)	.659	**−1.66 (0.48)**	**.005**
Polysubstance Use	−1.68 (1.73)	.443	**−6.56 (1.69)**	**< .001**	**−7.24 (1.77)**	**< .001**	−1.64 (2.08)	.536	−0.80 (2.05)	.732	**−2.19 (0.61)**	**< .001**
Suicidality	−1.51 (1.14)	.288	−1.95 (1.09)	.154	−1.12 (0.68)	.201	−0.21 (0.69)	.772	0.41 (0.88)	.698	−0.44 (0.20)	.055
Mastery	0.09 (0.07)	.273	**0.21 (0.06)**	**.005**	0.12 (0.07)	.127	**0.17 (0.07)**	**.041**	0.11 (0.07)	.221	**0.09 (0.02)**	**< .001**
General Health	−0.12 (0.16)	.536	−0.05 (0.14)	.768	0.18 (0.14)	.304	**0.35 (0.14)**	**.037**	0.04 (0.14)	.772	0.05 (0.05)	.440
**Resilience by Either Reporters**												
Intrusive Grief	**−0.36 (0.07)**	**< .001**	−0.13 (0.16)	.548	−0.11 (0.12)	.526	−0.29 (0.12)	.074	0.01 (0.11)	.967	**−0.13 (0.04)**	**.011**
Grief-related Social Detachment/Insecurity	−0.13 (0.09)	.276	−0.36 (0.18)	.131	−0.22 (0.12)	.188	**−0.40 (0.13)**	**.011**	−0.20 (0.17)	.358	**−0.15 (0.05)**	**.007**
Personal Growth Through Grief	**0.48 (0.12)**	**< .001**	**1.03 (0.24)**	**< .001**	0.32 (0.17)	.139	0.20 (0.15)	.325	−0.23 (0.19)	.358	**0.21 (0.05)**	**< .001**
Internalizing Problems	−0.88 (1.94)	.723	−7.40 (3.39)	.097	−3.14 (2.18)	.298	**−5.58 (2.14)**	**.036**	−0.30 (3.07)	.967	−1.86 (0.88)	.104
MDD Diagnosis	−0.35 (0.51)	.633	**−1.99 (0.61)**	**.007**	−0.65 (0.52)	.358	−0.34 (0.54)	.650	0.53 (0.64)	.548	−0.30 (0.19)	.276
Externalizing Problems	−2.36 (1.54)	.276	−4.47 (3.31)	.325	−3.17 (2.06)	.276	**−5.77 (2.07)**	**.021**	−1.13 (2.16)	.707	**−2.07 (0.74)**	**.021**
Polysubstance Use	−1.68 (1.73)	.474	−3.79 (3.69)	.445	−1.29 (2.54)	.707	−2.47 (2.3)	.423	0.88 (2.6)	.802	−0.98 (0.82)	.358
Suicidality	−1.51 (1.14)	.330	−1.76 (0.72)	.053	−1.07 (0.71)	.281	−0.41 (0.87)	.723	8.12 (0.58)	**< .001**	−0.48 (0.24)	.131
Mastery	0.09 (0.07)	.325	**0.29 (0.1)**	**.021**	0.11 (0.09)	.358	0.27 (0.08)	**< .001**	0.20 (0.11)	.177	**0.11 (0.03)**	**< .001**
General Health	−0.12 (0.16)	.557	0.07 (0.28)	.873	−0.01 (0.16)	.967	0.10 (0.17)	.682	0.11 (0.19)	.682	0.003 (0.07)	.967

*Note.* All outcome measures are based on youth self-reports, except for the measure of suicidality, which combines both youth self-report and key informant report.

All regression analyses controlled for youth age at parental death, youth sex, intervention condition, and cause of parental death.

Adjusted *p* < .05 are bolded.

#### Phase 3: Baseline prediction of multidomain resilience

We tested the relations between multidomain resilience scores and demographics, including youth age at parental death, youth sex, and cause of parental death. We included the intervention condition as a control variable. The intervention condition is not expected to affect latent classifications in a randomized trial; its effect would be to lower or raise the slope within each class trajectory (Brincks et al., 2018; Muthén et al., 2002). The count scores of the five-domain multidomain resilience scores for agreement across reporters and by either reporter were the dependent variables, respectively, using Poisson regression. We applied false discovery rate correction (FDR; Benjamini et al., 2001) across all the p-values to adjust multiple tests separately for predicting resilience scores across reporters and by either reporter. The missing data in Phases 3 and 4 were handled with the full information maximum likelihood method (Muthén & Muthén, 1998–2017).

#### Phase 4: Domain-specific resilience and multidomain resilience as predictors of outcomes 15-years post-baseline

We examined associations of grief, mental health, mastery and general health outcomes 15-years post-baseline with domain-specific and multidomain resilience scores. Linear regressions were used for continuous outcome variables and logistic regressions for dichotomous outcome variables. Covariates included youth age, youth sex, cause of parental death, and intervention condition. We applied FDR across all the *p*-values to adjust for multiple tests separately for predictions from domain-specific and multidomain resilience scores based on agreement across reporters (see Table 4, upper section) and either reporter (see Table 4, lower section).

Out of the 240 youth, 164 (68.3%) had complete data on all 10 outcomes. Missing data on single 15-year outcome measures ranged from 26.3 to 30%. Little’s MCAR test showed that 15-year outcomes data were not missing completely at random, X^2^(35) = 73.42, *p* = .0002. Independent sample t-tests and chi-square tests showed youth with (*n* = 76) or without missing data (*n* = 164) on 15-year outcomes did not significantly differ based on youth baseline age, time since parental death, youth sex, cause of death, or group assignment (*ps* > .05). Youth with missing data on 15-year MDD diagnosis were less likely to experience a parental death due to accident (X^2^(1) = 5.33, *p* = .02, Odds Ratio [OR] = 0.42). This indicated that the missingness could be associated with observed variables (i.e., Missing at Random). No other missingness on any single outcome variables was related to youth baseline age, time since parental death at baseline, youth sex, cause of death, or group assignment (*ps* > .05).

## Results

### Growth trajectory patterns

GMM for all 10 outcomes identified at least one resilient group. As shown in Table 1, significant variability in the proportion of youth classified in resilient groups on 10 *single outcomes* was found, ranging from 36.3% (*n* = 87) for youth-report intrusive grief to 83% (*n* = 198) for youth-report depression. With the exception of intrusive grief, resilience was the modal trajectory for the other nine variables as assessed by single outcomes, with a pooled prevalence of 62.3% (median = 62.1%) across 10 variables. The fit statistics, proportions, observed individual trajectories, and estimated mean trajectory with the growth parameters from the Mplus output in each class are presented in the supplemental materials.

Significant variability was found across the five domains in the proportion of youth classified as resilient, with the lowest rate being grief (36.3%). On average, 43.2% of youth (median = 44.6%) were classified as *domain-specific resilient* (i.e., resilient on a single domain) on a given domain based on across-reporter agreement, while 74.1% (median = 79.6%) were classified as *domain-specific resilient* on a given domain based on either-reporter coding. The internalizing problems domain showed the largest discrepancy, with 45% classified as resilient across reporters and 95.8% classified as resilient by either reporter. For externalizing problems, academic competence, and peer competence about one-third to half of youth were classified as resilient based on agreement across reporters, whereas more than three-quarters were classified as resilient by either reporter.

#### Domain 1: Grief

##### Intrusive grief, youth-report

**Figure 1.**
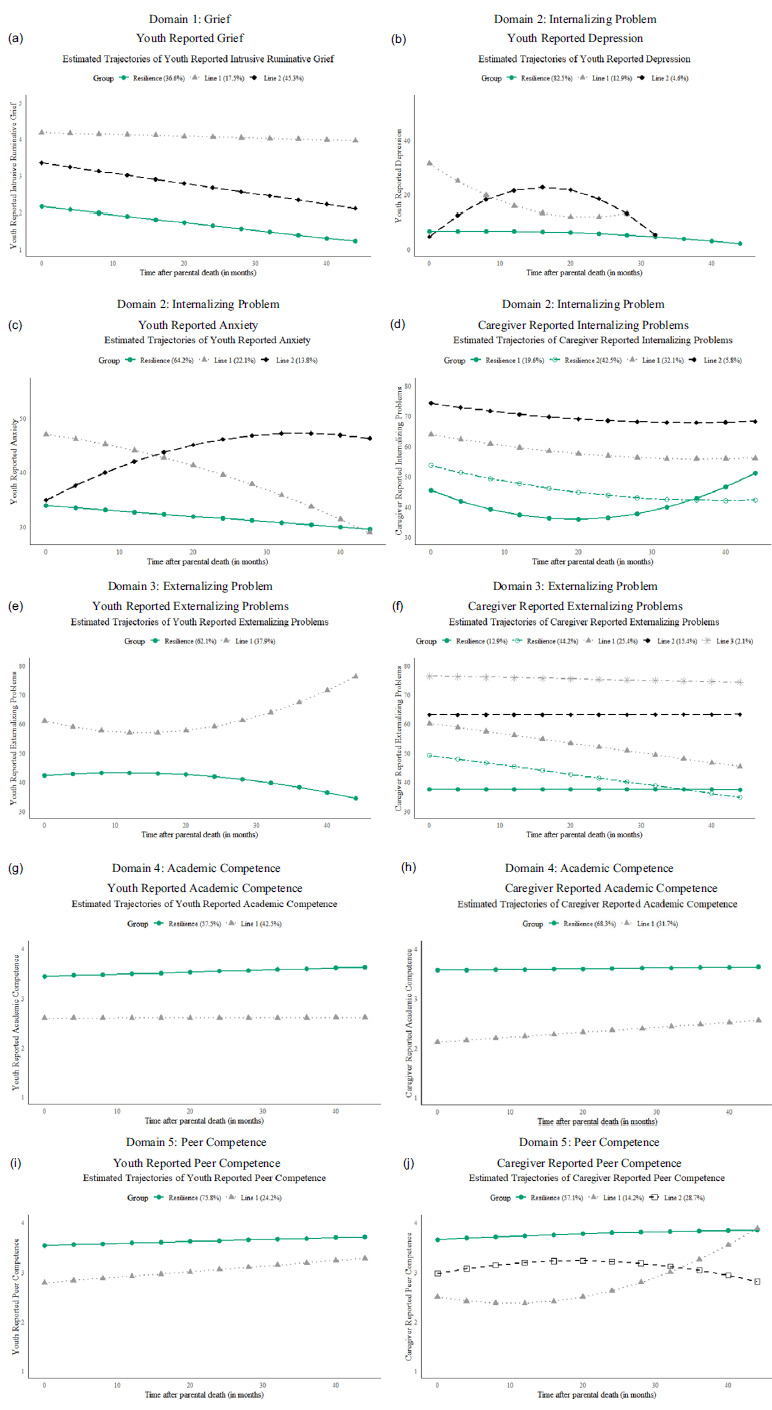
Growth mixture models illustrating the estimated means for each profile across all 10 variables.

Fit statistics for GMM and sample posterior probabilities in each latent class are shown in Table S1; the observed individual trajectories and mean trajectory for each class can be found in Figure S1; and the observed individual trajectories and estimated mean trajectory for each class are shown in Figure 1(a). The 3-class linear model was selected since it has lowest or near lowest BIC and saBIC, a significant LMR test, and highest entropy. A *resilient* (*n* = 87, 36%) group was identified, demonstrating lower than baseline mean (*M*_*W1*_ = 3.49, *SD*_*W1*_ = 0.86) of self-reported intrusive grief thoughts (i.e., occurrence ranged between not at all and less than once a week) across W1 – W3.

#### Domain 2: Internalizing problems

##### Depression, youth-report

The 3-class quadratic growth model (Figure 1(b); Table S2; Figure S2) provided the optimal solutions based on near lowest BIC, acceptable saBIC, and highest entropy. No LMR test was significant. A *resilient* group (*n* = 198, 82.5%) was identified, demonstrating lower than baseline mean (*M*_*W1*_ = 9.84, *SD*_*W1*_ = 7.55) across W1 – W3, which is lower than the clinical cutoff score of 16 (Timbremont et al., 2004) across W1 – W3.

##### Anxiety, youth-report

The 3-class quadratic growth model (Figure 1(c); Table S3; Figure S3) provided the optimal solutions based on acceptable BIC and saBIC and highest entropy. No LMR test was significant. A *resilience* group (*n* = 154, 64.2%) was identified, demonstrating lower than baseline mean (*M*_*W1*_ = 37.62, *SD*_*W1*_ = 6.88) across W1 – W3. It is noteworthy that it is also lower than the clinical cutoff of 38 (Stallard et al., 2001) across W1 – W3.

##### Internalizing problems, caregiver-report

The 4-class quadratic growth model (Figure 1(d); Table S4; Figure S4) provided the optimal solution based on near lowest BIC and saBIC, significant LMR test, and high entropy. Two *resilience* groups were identified (*n* = 149, 62.1%), demonstrating lower than baseline mean (*M*_*W1*_ = 54.91, *SD*_*W1*_ = 10.87) and below the marginal clinical level across W1 – W3.

#### Domain 3: Externalizing problems

##### Externalizing problems, youth-report

The 2-class quadratic growth model (Figure 1(e); Table S5; Figure S5) provided the optimal solution based on near lowest BIC and saBIC, highest entropy, and significant LMR test. One *resilient* group (*n* = 149, 62.1%) was identified, demonstrating lower than baseline mean (*M*_*W1*_ = 51.06, *SD*_*W1*_ = 11) across W1 – W3.

##### Externalizing problems, caregiver-report

The 4-class linear growth model provided the optimal solution based on lowest or near lowest BIC and saBIC, significant LMR test, and highest entropy (Figure 1(f); Table S6; Figure S6). Two *resilient* groups were identified (*n* = 137 combined across the two groups, 57.1%), demonstrating lower than baseline mean (*M*_*W1*_ = 53.38, *SD*_*W1*_ = 10.68) and below the marginal clinical level from W1 – W3.

#### Domain 4: Academic competence

##### Academic competence, youth-report

The 2-class linear growth model (Figure 1(g); Table S7; Figure S7) provided the optimal solutions based on acceptable BIC and saBIC, significant LMR test, and highest entropy. Additionally, a comparison of the 2-class linear versus 2-class quadratic models indicated that the quadratic term was not significant in any of the two classes. A *resilient* (*n* = 138, 57.5%) group was identified, demonstrating higher than baseline mean (*M*_*W1*_ = 3.05, *SD*_*W1*_ = 0.64) across W1 – W3.

##### Academic competence, caregiver-report

The 2-class linear growth model (Figure 1(h); Table S8; Figure S8) provided the optimal solutions based on acceptable BIC and saBIC, significant LMR test, and near highest entropy. Additionally, a comparison of the 2-class linear versus 2-class quadratic models indicated that the quadratic term was not significant in any of the two classes. A *resilient* group (*n* = 164, 68.3%) was identified, demonstrating higher than baseline mean (*M*_*W1*_ = 3.09, *SD*_*W1*_ = 0.73) across W1 – W3.

#### Domain 5: Peer competence

##### Peer competence, youth-report

The 2-class linear growth model (Figure 1(i); Table S9; Figure S9) provided the optimal solutions based on acceptable BIC and saBIC, significant LMR test, and near highest entropy. Additionally, a comparison of the 2-class linear versus 2-class quadratic models indicated that the quadratic term was not significant in any of the two classes. A *resilient* group (*n* = 182, 75.8%) was identified, demonstrating higher than baseline mean (*M*_*W1*_ = 3.36, *SD*_*W1*_ = 0.49) across W1 – W3.

##### Peer competence, caregiver-report

The 3-class quadratic growth model (Figure 1(j); Table S10; Figure S10) provided the optimal solutions based on acceptable BIC and saBIC, significant LMR test, and acceptable entropy. A *resilient* group (*n* = 137, 57.1%) was identified, demonstrating higher than baseline mean (*M*_*W1*_ = 3.30, *SD*_*W1*_ = 0.5) across W1 – W3.

### Multidomain resilience

**Figure 2.**
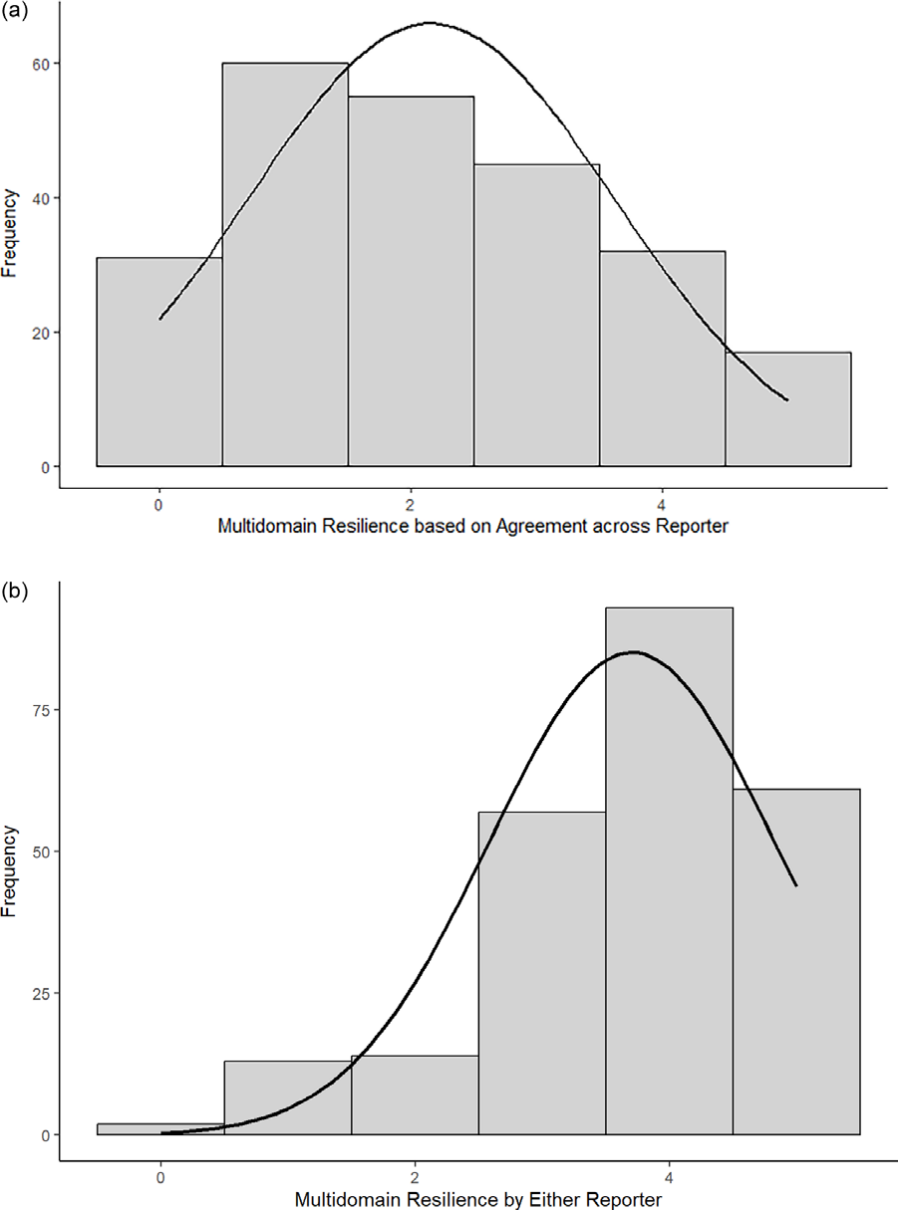
Distribution of multidomain resilience scores across reporters (a, top) and by either reporter (b, bottom). *Note*. Although the distribution does not meet the statistical criteria for normality, we have included normal distribution curves (shown in black) in the figure for reference. This is to illustrate that the observed distribution does not strongly deviate from a normal distribution.

We assessed multidomain resilience as the count of the number of the five domains in which the youth were classified as resilient. Table 2 presents the frequency of the multidomain resilience scores based on agreement across reporters and by either reporter. Using the criteria of resilient by both reporters, on average, youth exhibited across-reporter classification as resilient on 2.16 (*SD* = 1.45) domains (median = 2, skewness = 0.29, kurtosis = −0.86; Figure 2(a)). Only 17 youth (7.1%) were classified as resilient across all five domains, and 31 youth (12.9%) were classified as non-resilient across all five domains. For resilience by either reporter, youth were classified as resilient by either reporter on an average of 3.70 (*SD* = 1.12) domains (median = 4, skewness = −0.92, kurtosis = 0.59; Figure 2(b)). Sixty-one youth (25.4%) were classified as resilient across all five domains, and two youth (0.8%) were classified as non-resilient across all five domains by either reporter.

### Baseline predictors of multidomain resilience

As shown in Table 3, none of the demographic or intervention condition variables significantly predicted multidomain resilience scores based on agreement across reporters and by either reporter after FDR adjustment.

### Domain-specific and multidomain resilience as predictors of 15-year outcomes

The upper part of Table 4 presents results of predicting 15-year outcomes from the domain-specific resilient and multidomain resilient scores, where the resilient classification within each domain was based on agreement across reporters. Resilience in grief was associated with two grief-related 15-year outcomes, one indicating intrusive grief thoughts and the other indicating positive growth from grief. Resilience in internalizing problems and academic competence each predicted multiple measures of problem and positive functioning at the 15-year outcomes. Resilience in externalizing problems was predictive of lower grief-related social detachment/insecurity, externalizing problems, and polysubstance use. Resilience in peer competence did not predict any problem or positive functioning outcomes. Multidomain resilience, as reported by both reporters, predicted a broad range of grief, mental health, and mastery outcomes. Although no longer statistically significant after FDR correction (*p* = .055), the association between multidomain resilience and suicidality may be of interest for further investigation.

The lower part of Table 4 presents results of predicting outcomes using the criterion of being classified as resilient by either reporter. The predictions of 15-year outcomes by resilience in grief were the same since both methods of assessing the grief resilience domain only used youth report. Resilience in peer competence only predicted one of the 10 outcomes. Domain-specific resilience on internalizing problems, externalizing problems, academic competence, and multidomain resilience as classified by either reporter predicted somewhat fewer 15-year problem and positive functioning variables as compared to resilience as classified across reporters.

## Discussion

The current study adds to prior literature on multidimensional resilience (Luthar et al., 2000) and the interrelations of domains of functioning across development (Masten & Cicchetti, 2010) by describing the prevalence of problem and competence resilient trajectories across multiple domains, the distribution of cumulative resilience across domains, and the predictive relations between these resilience domains and outcomes 15-years post-baseline in a sample of parentally bereaved youth. The three main findings from this research are that bereaved youth experience differential prevalence of resilient trajectories across different domains in which they are at risk, that few bereaved youth are resilient across all such domains or have no domain on which they are resilient, and that resilient trajectories in specific domains differentially predict outcomes 15 years following baseline assessment. These findings are discussed in terms of their implications for estimating the prevalence of resilience, how understanding theoretical processes underlying resilience can inform the design of interventions to promote it across domains of functioning, and the study’s limitations and directions for future research on resilience among youth exposed to major life adversities.

An important advance in resilience assessment has been the estimation of trajectories of outcomes over repeated observations over time using growth mixture modeling (GMM; e.g., Bonanno & Diminich, 2013). Prior estimates of resilience prevalence using GMM concluded that resilience is very common based on multiple studies of resilient trajectories of functioning on a single outcome (Galatzer-Levy et al., 2018). Others have argued that resilience is rare when assessed across multiple outcome following adversity (Infurna & Luthar, 2017a; 2017b). Our findings are consistent with the conclusion, that resilience is indeed uncommon, if assessed as a dichotomous variable across all five domains in which bereaved youth are at risk (i.e. multidomain resilience). Only 7.1% of youth met the criteria for resilience across all five domains based on agreement across reporters, and only 25% met the criteria for resilience based on either reporter. Yet very few youth were non-resilient across domains. Only 12.9% of youth did not meet criteria for resilience based on agreement across reporters and 0.8% of youth did not meet criteria for resilience by either reporter. Overall, cumulative multidomain resilience following the major adversity of parental bereavement followed a near-normal distribution, suggesting that most youth adapt well in some domains but not others, and that few youth have no area of resilience nor are resilient across all domains. Accordingly, multidomain resilience is better understood as a count of the number of discrete domains on which youth are resilient rather than a dichotomous phenomenon requiring resilience on all domains for which youth are at risk. To further unpack nuances and complexity of cross-domain resilience, future research could use person-centered approaches, such as latent profile analysis, to identify common patterns of resilient and non-resilient domains, which may reveal qualitatively distinct resilience profiles with different implications for intervention and long-term outcomes.

An important methodological strength of the current study is to assess resilience within domains based on agreement across multiple reporters’ resilient trajectories. Drawing from the multi-informant assessment literature (e.g., Achenbach et al., 1987; De Los Reyes et al., 2015), we view the cross-reporter agreement resilience as a more stringent criterion than single reporter resilience, as it reflects consensus of observation across distinct contexts. Cross-reporters resilience may indicate generalized or cross-context resilience, while resilience reported by only one informant may reflect resilience as observed within a specific context. Prior scholarship has also emphasized that ratings from others can be especially valuable in resilience research because they capture competencies in salient developmental domains more reliably than self-reports alone (Infurna & Luthar, 2018; Luthar & Zelazo, 2003; Luthar, 2015). However, we present prevalence estimates for both methods to allow comparisons with prior studies that have used only a single reporter to assess trajectories of functioning over time. Not surprisingly resilience prevalence was much higher when based on either single report compared to the more stringent criterion of agreement across reporters. We also acknowledge that differences between reporters are shaped by the combination of distinct characteristics and contexts in which they observe children’s behavior (e.g., Bauer et al., 2013; Cai et al., 2025). Future research should aim to disentangle rater bias from meaningful contextual variation, in order to better understand the sources and implications of informant discrepancies in resilience research.

Consistent with prior research (see Luthar, 2015 for a review of studies on diversity of resilience across domains), we also find that resilience prevalence differs across different domains of problem and competent functioning. There was a broad range of resilient trajectories across five domains when assessed by agreement across reporters (36.3% to 48.3%; *M =* 2.16; median = 2) and by either reporter (36.3% to 91.3%; *M =* 3.70; median = 4). Similar to Galatzer-Levy et al., 2018, these findings indicate that on some specific domains and reporters, resilience is common. Domains on which bereaved youth are resilient may provide them resources to adapt following the death. Those who were not resilient on any domain of functioning (12.9% based on agreement across reporters), are likely at increased risk for long-term difficulties of adaptation. Our conclusion is that there is no single estimate of resilience prevalence that does justice to the variety of outcomes across domains for bereaved youth. Rather, the variety of outcomes reflects a dynamic process in which adaptive processes (Masten, 2014) are more or less successful in impacting different areas of functioning. The lack of associations between demographic and death-related factors and resilience may reflect that these predictors do not capture the underlying processes that lead to resilience.

We agree with Infurna and Luthar (2016b) that estimates of resilience prevalence have consequences for the allocation of resources to treat or prevent problem outcomes following adversity. If resilience is seen as common, then interventions to promote resilience may not be needed for the great majority of people (e.g., Bonanno et al., 2011), and intervention resources should focus only on the few who are at risk of suffering. Our finding that most youth show resilience in some domains but struggle in others suggests that interventions should target the specific domains in which bereaved youth face difficulties, particularly those linked to long-term adverse outcomes.

Our study found that some domains of resilience, including academic competence and internalizing problems, as well as multidomain resilience predicted multiple outcomes 15-years post-baseline, whereas resilience on the grief domain had a specific effect to predict only long-term grief outcomes. We will focus our discussion on those findings that have the strongest implications for interventions and the underlying processes that may be the most productive targets for interventions.

Luthar et al. (2000) propose that resilience-promoting interventions should target the processes that drive positive or problematic outcomes. Research has identified empirically supported protective individual-, family-, and community-level processes that are associated with resilience of bereaved youth (Alvis et al., 2022; Hoppe et al., 2024; Luthar et al., 2015; Sandler et al., 2007; Sandler et al., 2024; Tein et al., 2006). Overall, the interactions of individual-, family-, and community-level processes shape youths’ perceptions of their world as supportive or threatening, influencing both their sense of self (e.g., internalizing problems) and their engagement with their environments (e.g., family and school). Interventions that target malleable processes within and across these domains offer proximal leverage points for strengthening resilience among bereaved youth. For example, the finding that resilience in the grief domain predicts lower intrusive grief and higher growth through grief 15-years post-baseline supports the long-term benefit of early interventions to promote adaptive grief. At the individual level, these interventions may focus on psychoeducation to normalize grief, receiving the support from others who are grieving, and promoting effective grief focused coping and emotion regulation skills (e.g. Hill et al., 2019; Sandler et al., 2010). In addition, interventions could also be designed to promote family level processes that are related to intrusive grief such as caregiver facilitation of children’s grief discussion (Alvis et al., 2022; Hoppe et al., 2025).

Our finding that resilience on internalizing problems and academic competence domains predict multiple mental health, grief, and mastery outcomes indicates that multi-level interventions to promote processes that account for resilience on these domains may have long-term impacts on functioning across multiple domains of problem and positive functioning. For example, a multi-component program to teach youth effective coping and emotion regulation skills, and to teach caregivers effective parenting and grief facilitation skills had positive impact to reduce internalizing problems including major depression (Tein et al., 2006; Sandler et al., 2024) and improve academic outcomes (Schoenfelder et al., 2015). In a study using a nationally representative sample, Oosterhoff et al. (2018) found that bereavement due to sudden death was associated with lower academic functioning, weaker beliefs that teachers are fair, reduced school belongingness and enjoyment, and greater concentration difficulties compared to non-bereaved peers. The authors as well as others recommended school-based programs, including teacher training and academic tutoring, to support the academic success of bereaved youth.

The current study had multiple limitations. The study adopted a conceptualization of resilient trajectories as consistently lower than expected levels of problems or higher levels of competent functioning (Luthar et al., 2015; Rutter, 2012). This conceptualization aligns with prior research on resilience using a GMM approach (Bonanno, 2004), including a meta-analysis of 54 studies with children and adults (Galatzer-Levy et al., 2018) and was the focus of an ongoing debate in the literature regarding the prevalence of resilience (e.g., 2016b, Galatzer-Levy & Bonanno, 2016; Infurna & Luthar, 2016a). However, we acknowledge that other trajectories, including those reflecting recovery of positive functioning over time, should also be considered indicators of resilience. The prevalence and the correlates of recovery trajectories in different domains is an important area of future research.

The current study investigates resilience of youth who experienced a specific major adversity, the death of a parent. Further research is needed to assess whether the findings concerning domains of resilience generalize across other acute adversities such as parental divorce, accidents, or exposure to violence, as well as to more chronic adversities, such as growing up in poverty. Moreover, and although this is not a limitation per se, it should be noted that the study investigated resilience in terms of domains of outcomes, rather than the processes or mechanisms underlying these outcomes. Future research should study these underlying mechanisms and explore how they lead to domain-specific resilience or resilience across multiple domains of functioning.

Although assessing resilience using a multidomain approach based on the GMM derived trajectories provides a useful perspective on resilience, it is important to not overinterpret the meaning of the individual trajectories. GMM is an exploratory analysis tool and using it to approximate trajectories is subject to a certain degree of uncertainty and may differ depending on the assumptions specified in the model (Infurna & Grimm, 2018). To address common convergence issues with limited sample sizes, we constrained most variances of the growth factors (i.e., intercept, linear slope, quadratic slope) to zero (Hox, 2002; Nagin, 1999) which might have affected the class solutions or parameter estimates. This underscores the importance of replicating the study with large samples. Furthermore, we decided the optimal solutions based on a combination of criteria since there are no definitive rules for selecting the best model (Hair & Black, 2000; Ram & Grimm, 2009) and assigned each youth to the most likely trajectory including the resilience group for each measure. More studies of multidomain resilience in youth are needed with other samples of bereaved youth and those experiencing other adversities to replicate and extend the findings from this study. In addition, future research should move beyond the study of individual domains to explore a configural approach to identify patterns of resilient domains for which youth are at risk. Methods such as latent class analysis may illuminate these cross-domain resilience patterns and clarify which patterns of resilience are associated with what long-term problems and competencies over time.

Despite these limitations, the study highlights the value of a multidomain approach for understanding the prevalence of resilience and the associations between domain-specific and multidomain resilience with long-term outcomes among youth who have experienced one of the most stressful adversities, the death of a parent (Yamamoto et al., 1996; McKay et al., 2022). The findings significantly contribute to our understanding of the challenges youth encounter as they adapt following a major adversity and directions for the development of future research to inform the development of interventions to promote their healthy, satisfying and productive lives.

## Supporting information

10.1017/S0954579425101107.sm001 Sandler et al. supplementary material Sandler et al. supplementary material

## Data Availability

The analysis code and materials necessary to reproduce the analysis and replicate the findings are available from the first authors upon reasonable request.

## Supplementary material

The supplementary material for this article can be found at https://doi.org/10.1017/S0954579425101107.

## AI

AI was not used in the development or writing of this paper.

## Pre-registration

We did not qualify for pre-registration because the growth trajectories of one variable had been previously analyzed in a separate published study described in this paper.
