# Supplementary material for: Domain-specific and multidomain resilience among parentally bereaved youth: Assessment and associations with long-term outcomes
Source: Dev Psychopathol. 2026 Feb 10:1–15. Online ahead of print. doi: 10.1017/S0954579425101107 (PMC12931431; doi:10.1017/S0954579425101107)
Supplement: Sandler et al. supplementary material [file S0954579425101107sup001.docx]

**Supplementary Material 1 Tables**

Table S1. Fit statistics for GMM of youth reported of intrusive grief from Wave 1 to Wave 3 with 1- to 5-class solutions and sample posterior probabilities in each latent class.

|  | **Fit indices** | | | | **Class Proportion** | | | | |
| --- | --- | --- | --- | --- | --- | --- | --- | --- | --- |
| **Number of classes** | **BIC** | **SaBIC** | LMR  *p*-value | entropy | **1** | **2** | **3** | **4** | **5** |
| **Linear** |  |  |  |  |  |  |  |  |  |
| 1 | 1976.84 | 1938.80 |  |  |  |  |  |  |  |
| 2 | 1785.03 | 1737.49 | .01 | .74 | .48 | .52 |  |  |  |
| **3**^a^ | **1711.11** | **1654.06** | **<.001** | **.80** | **.46** | **.18** | **.36^b^** |  |  |
|  |  |  |  |  | **(*n =* 111)** | **(*n =* 42)** | **(*n =* 87)** |  |  |
| 4 | 1717.31 | 1650.74 | .06 | .78 | .12 | .36 | .48 | .04 |  |
| 5 | 1723.80 | 1647.72 | .11 | .79 | .45 | .12 | .35 | .05 | .02 |
|  |  |  |  |  |  |  |  |  |  |
| **Quadratic** |  |  |  |  |  |  |  |  |  |
| 1 | 1981.14 | 1939.93 |  |  |  |  |  |  |  |
| 2 | 1795.93 | 1742.04 | .02 | .74 | .47 | .53 |  |  |  |
| 3 | 1726.79 | 1660.22 | <.001 | .80 | .17 | .47 | .36 |  |  |
| 4 | 1734.06 | 1654.82 | .54 | .73 | .12 | .40 | .17 | .31 |  |
| 5 | 1742.91 | 1650.98 | .36 | .72 | .09 | .43 | .15 | .26 | .08 |

^a^ The 3-class linear model was chosen since the 3-class linear model has significant Lo-Mendell-Rubin (LMR) test, highest entropy, lowest Bayesian Information Criterion (BIC), and comparable sample-adjusted BIC (SaBIC). Additionally, a comparison of the 3-class linear versus 3-class quadratic models indicated that the quadratic term was not significant in any of the three classes.

^b^ The resilience group.

Table S2. Fit statistics for GMM of youth reported depression from Wave 1 to Wave 3 with 1- to 5-class solutions and sample posterior probabilities in each latent class.

|  | **Fit indices** | | | | **Class Proportion** | | | | |
| --- | --- | --- | --- | --- | --- | --- | --- | --- | --- |
| **Number of classes** | **BIC** | **SaBIC** | LMR  *p*-value | entropy | **1** | **2** | **3** | **4** | **5** |
| **Linear** |  |  |  |  |  |  |  |  |  |
| 1 | -42.80 | -87.17 |  |  |  |  |  |  |  |
| 2 | -103.62 | -157.51 | .23 | .88 | .89 | .11 |  |  |  |
| 3 | -134.21 | -197.61 | .17 | .84 | .79 | .11 | .10 |  |  |
| 4 | -138.30 | -208.03 | .13 | .86 | .13 | .10 | .01 | .75 |  |
| 5 | -125.88 | -208.30 | .41 | .85 | .07 | .02 | .11 | .06 | .74 |
|  |  |  |  |  |  |  |  |  |  |
| **Quadratic** |  |  |  |  |  |  |  |  |  |
| 1 | -37.32 | -84.87 |  |  |  |  |  |  |  |
| 2 | -99.00 | -159.22 | .17 | .85 | .12 | .88 |  |  |  |
| **3**^a^ | **-128.34** | **-201.24** | **.24** | **.88** | **.13**  **(*n =* 31)** | **.05**  **(*n =* 11)** | **.83^b^**  **(n =198)** |  |  |
| 4 | -120.75 | -206.33 | .62 | .82 | .09 | .12 | .76 | .03 |  |
| 5 | -121.28 | -216.37 | .65 | .85 | .11 | .04 | .20 | .60 | .05 |

^a^ The 3-class quadratic model was chosen based on the highest entropy, lowest Bayesian Information Criterion (BIC), and comparable sample-adjusted BIC (SaBIC). No Lo-Mendell-Rubin (LMR) test was significant.

^b^ The resilience group.

Table S3. Fit statistics for GMM of youth reported anxiety from Wave 1 to Wave 3 with 1- to 5-class solutions and sample posterior probabilities in each latent class.

|  | **Fit indices** | | | | **Class Proportion** | | | | |
| --- | --- | --- | --- | --- | --- | --- | --- | --- | --- |
| **Number of classes** | **BIC** | **SaBIC** | LMR  *p*-value | entropy | **1** | **2** | **3** | **4** | **5** |
| **Linear** |  |  |  |  |  |  |  |  |  |
| 1 | -42.17 | -86.55 |  |  |  |  |  |  |  |
| 2 | -43.49 | -97.38 | .35 | .62 | .10 | .90 |  |  |  |
| 3 | -81.51 | -141.73 | .30 | .78 | .63 | .18 | .19 |  |  |
| 4 | -89.71 | -159.45 | .60 | .74 | .18 | .52 | .17 | .14 |  |
| 5 | -89.20 | -168.45 | .10 | .75 | .50 | .12 | .19 | .15 | .05 |
|  |  |  |  |  |  |  |  |  |  |
| **Quadratic** |  |  |  |  |  |  |  |  |  |
| 1 | -37.05 | -84.60 |  |  |  |  |  |  |  |
| 2 | -32.47 | -92.69 | .69 | .60 | .17 | .83 |  |  |  |
| **3^a^** | **-67.82** | **-137.56** | **.15** | **.79** | **.14**  **(*n =* 33)** | **.64^b^**  **(*n =* 154)** | **.22**  **(*n =* 53)** |  |  |
| 4 | -75.76 | -158.18 | .12 | .75 | .20 | .50 | .13 | .17 |  |
| 5 | -74.93 | -170.02 | .62 | .76 | .18 | .03 | .48 | .16 | .15 |

^a^ The 3-class quadratic model was chosen based on the highest entropy, lowest Bayesian Information Criterion (BIC) and comparable sample-adjusted BIC (SaBIC). No Lo-Mendell-Rubin (LMR) test was significant.

^b^ The resilience group.

Table S4. Fit statistics for GMM of caregiver reported internalizing problems from Wave 1 to Wave 3 with 1- to 5-class solutions and sample posterior probabilities in each latent class.

|  | **Fit indices** | | | | **Class Proportion** | | | | | |
| --- | --- | --- | --- | --- | --- | --- | --- | --- | --- | --- |
| **Number of classes** | **BIC** | **SaBIC** | **LMR p** | LMR  *p*-value | **1** | **2** | **3** | **4** | **5** | **6** |
| **Linear** |  |  |  |  |  |  |  |  |  |  |
| 1 | 5265.20 | 5223.99 |  |  |  |  |  |  |  |  |
| 2 | 5050.71 | 4999.99 | <.001 | .83 | .38 | .62 |  |  |  |  |
| 3 | 5030.22 | 4970.00 | .20 | .70 | .25 | .45 | .30 |  |  |  |
| 4 | 5015.55 | 4945.81 | .01 | .77 | .06 | .45 | .19 | .30 |  |  |
| **5** | 5017.59 | 4938.35 | 0.01 | .81 | .00 | .20 | .01 | .44 | .31 |  |
| 6 | 5030.27 | 4941.51 | 0.38 | .77 | .20 | .09 | .26 | .43 | .00 | .03 |
|  |  |  |  |  |  |  |  |  |  |  |
| **Quadratic** |  |  |  |  |  |  |  |  |  |  |
| 1 | 4992.62 | 4945.07 |  |  |  |  |  |  |  |  |
| **2** | 5050.77 | 4993.71 | <.001 | .84 | .38 | .63 |  |  |  |  |
| 3 | 5034.67 | 4964.93 | .43 | .72 | .45 | .32 | .24 |  |  |  |
| **4** ^a^ | **5019.61** | **4937.20** | **<.001** | **.78** | **.32**  **(*n* = 77)** | **.20** ^b^  **(*n* = 47)** | **.43** ^b^  **(*n* = 102)** | **.06**  **(*n* = 14)** |  |  |
| 5 | 5026.11 | 4931.02 | .01 | .81 | .20 | .43 | .32 | .00 | .05 |  |
| 6 | 5036.26 | 4928.49 | .56 | .75 | .09 | .19 | .08 | .00 | .22 | .42 |

^a^ The 3-class quadratic model was chosen based on the significant Lo-Mendell-Rubin (LMR) test, high entropy, and near lowest Bayesian Information Criterion (BIC) as well as sample-adjusted BIC (SaBIC).

^b^ The resilience groups.

Table S5. Fit statistics for GMM of youth reported externalizing problems from Wave 1 to Wave 3 with 1- to 5-class solutions and sample posterior probabilities in each latent class.

|  | **Fit indices** | | | | **Class Proportion** | | | | | |
| --- | --- | --- | --- | --- | --- | --- | --- | --- | --- | --- |
| **Number of classes** | **BIC** | **SaBIC** | **LMR p** | LMR  *p*-value | **1** | **2** | **3** | **4** | **5** | **6** |
| **Linear** |  |  |  |  |  |  |  |  |  |  |
| 1 | 5292.55 | 5337.80 |  |  |  |  |  |  |  |  |
| 2 | 5195.32 | 5144.61 | .001 | .71 | .40 | .60 |  |  |  |  |
| 3 | 5164.35 | 5104.12 | .03 | .70 | .14 | .48 | .38 |  |  |  |
| 4 | 5164.79 | 5095.06 | .15 | .64 | .18 | .07 | .28 | .46 |  |  |
| **5** | 5170.63 | 5091.38 | .40 | .71 | .48 | .02 | .19 | .05 | .27 |  |
| 6 | 5164.24 | 5075.49 | .34 | .67 | .13 | .02 | .34 | .32 | .14 | .07 |
|  |  |  |  |  |  |  |  |  |  |  |
| **Quadratic** |  |  |  |  |  |  |  |  |  |  |
| 1 | 5343.26 | 5298.89 |  |  |  |  |  |  |  |  |
| **2 ^a^** | **5195.85** | **5138.79** | **<.001** | **.73** | **.62** ^b^  **(*n* = 149)** | **.38**  **(*n* = 91)** |  |  |  |  |
| 3 | 5169.12 | 5099.39 | .10 | .68 | .17 | .46 | .37 |  |  |  |
| 4 | 5179.61 | 5097.19 | .15 | .70 | .48 | .32 | .15 | .05 |  |  |
| 5 | 5186.74 | 5091.64 | .38 | .66 | .36 | .30 | .06 | .10 | .18 |  |
| 6 | 5197.55 | 5089.78 | .83 | .68 | .12 | .05 | .37 | .06 | .14 | .27 |

^a^ The 2-class quadratic model was chosen based on the highest entropy, and comparable Bayesian Information Criterion (BIC) as well as sample-adjusted BIC (SaBIC). No Lo-Mendell-Rubin (LMR) test was significant.

^b^ The resilience group.

Table S6. Fit statistics for GMM of caregiver reported externalizing problems from Wave 1 to Wave 3 with 1- to 5-class solutions and sample posterior probabilities in each latent class.

|  | **Fit indices** | | | | **Class Proportion** | | | | | |
| --- | --- | --- | --- | --- | --- | --- | --- | --- | --- | --- |
| **Number of classes** | **BIC** | **SaBIC** | **LMR p** | LMR  *p*-value | **1** | **2** | **3** | **4** | **5** | **6** |
| **Linear** |  |  |  |  |  |  |  |  |  |  |
| 1 | 5248.22 | 5207.02 |  |  |  |  |  |  |  |  |
| 2 | 5002.57 | 4951.85 | <.001 | .85 | .60 | .40 |  |  |  |  |
| 3 | 4963.62 | 4903.40 | .49 | .73 | .41 | .36 | .23 |  |  |  |
| 4 | 4949.00 | 4879.27 | .75 | .77 | .23 | .42 | .05 | .30 |  |  |
| **5 ^a^** | **4942.79** | **4863.54** | **<.001** | **.76** | **.02**  **(*n* = 5)** | **.25**  **(*n* = 61)** | **.15**  **(*n* = 37)** | **.13** ^b^  **(*n* = 31)** | **.44** ^b^  **(*n* = 106)** |  |
| 6 | 4945.71 | 4856.96 | .64 | .69 | .02 | .06 | .20 | .22 | .32 | .18 |
|  |  |  |  |  |  |  |  |  |  |  |
| **Quadratic** |  |  |  |  |  |  |  |  |  |  |
| 1 | 5253.66 | 5209.29 |  |  |  |  |  |  |  |  |
| 2 | 5005.22 | 4948.17 | <.001 | .85 | .60 | .40 |  |  |  |  |
| 3 | 4977.48 | 4907.75 | .56 | .72 | .39 | .24 | .37 |  |  |  |
| 4 | 4964.46 | 4882.05 | .17 | .77 | .06 | .17 | .33 | .44 |  |  |
| 5 | 4966.33 | 4871.23 | .17 | .76 | .02 | .24 | .17 | .14 | .43 |  |

^a^ The 5-class linear model was chosen based on the significant Lo-Mendell-Rubin (LMR) test, near lowest Bayesian Information Criterion (BIC) and sample-adjusted BIC (SaBIC), and comparable entropy.

^b^ The resilience groups.

Table S7. Fit statistics for GMM of youth reported academic competence from Wave 1 to Wave 3 with 1- to 5-class solutions and sample posterior probabilities in each latent class.

|  | **Fit indices** | | | | **Class Proportion** | | | | |
| --- | --- | --- | --- | --- | --- | --- | --- | --- | --- |
| **Number of classes** | **BIC** | **SaBIC** | LMR  *p*-value | entropy | **1** | **2** | **3** | **4** | **5** |
| **Linear** |  |  |  |  |  |  |  |  |  |
| 1 | 1436.46 | 1395.25 |  |  |  |  |  |  |  |
| **2^a^** | **1297.75** | **1247.03** | **<.001** | **.75** | **.58^b^**  **(*n =* 138)** | **.42**  **(*n =* 102)** |  |  |  |
| 3 | 1287.18 | 1226.96 | .10 | .72 | .38 | .12 | .5 |  |  |
| 4 | 1284.22 | 1214.48 | .08 | .72 | .36 | .47 | .04 | .12 |  |
| 5 | 1291.43 | 1212.18 | .76 | .64 | .47 | .26 | .03 | .16 | .1 |
|  |  |  |  |  |  |  |  |  |  |
| **Quadratic** |  |  |  |  |  |  |  |  |  |
| 1 | 1441.55 | 1397.17 |  |  |  |  |  |  |  |
| 2 | 1307.95 | 1250.89 | <.001 | .75 | .44 | .56 |  |  |  |
| 3 | 1293.62 | 1223.89 | .09 | .73 | .12 | .50 | .38 |  |  |
| 4 | 1293.81 | 1211.39 | .72 | .63 | .34 | .43 | .1 | .13 |  |
| 5 | 1295.36 | 1200.27 | .37 | .63 | .41 | .12 | .26 | .05 | .15 |

^a^ The 2-class linear model was chosen based on significant Lo-Mendell-Rubin (LMR) test, highest entropy, and comparable Bayesian Information Criterion (BIC) as well as sample-adjusted BIC (SaBIC). Additionally, a comparison of the 2-class linear versus 2-class quadratic models indicated that the quadratic term was not significant in any of the two classes.

^b^ The resilience group.

Table S8. Fit statistics for GMM of caregiver reported academic competence from Wave 1 to Wave 3 with 1- to 5-class solutions and sample posterior probabilities in each latent class.

|  | **Fit indices** | | | | **Class Proportion** | | | | |
| --- | --- | --- | --- | --- | --- | --- | --- | --- | --- |
| **Number of classes** | **BIC** | **SaBIC** | LMR  *p*-value | entropy | **1** | **2** | **3** | **4** | **5** |
| **Linear** |  |  |  |  |  |  |  |  |  |
| 1 | 1754.11 | 1712.90 |  |  |  |  |  |  |  |
| **2^a^** | **1503.41** | **1452.69** | **<.001** | **.86** | **.32**  **(*n =* 76)** | **.68^b^**  **(*n =* 164)** |  |  |  |
| 3 | 1452.33 | 1392.10 | .14 | .81 | .15 | .36 | .49 |  |  |
| 4 | 1446.11 | 1376.38 | .14 | .81 | .32 | .50 | .05 | .13 |  |
| 5 | 1431.07 | 1351.82 | .21 | .87 | .42 | .14 | .31 | .08 | .05 |
|  |  |  |  |  |  |  |  |  |  |
| **Quadratic** |  |  |  |  |  |  |  |  |  |
| 1 | 1759.47 | 1715.09 |  |  |  |  |  |  |  |
| 2 | 1511.14 | 1454.08 | <.001 | .86 | .67 | .33 |  |  |  |
| **3** | 1463.79 | 1394.05 | .22 | .83 | .14 | .35 | .50 |  |  |
| 4 | 1452.58 | 1370.16 | .31 | .77 | .13 | .16 | .27 | .44 |  |
| 5 | 1437.53 | 1342.43 | .15 | .79 | .08 | .16 | .25 | .42 | .09 |

^a^ The 2-class linear model was chosen based on significant Lo-Mendell-Rubin (LMR) test, high entropy, and comparable Bayesian Information Criterion (BIC) as well as sample-adjusted BIC. Additionally, a comparison of the 2-class linear versus 2-class quadratic models indicated that the quadratic term was not significant in any of the two classes.

^b^ The resilience group.

Table S9. Fit statistics for GMM of youth reported peer competence from Wave 1 to Wave 3 with 1- to 5-class solutions and sample posterior probabilities in each latent class.

|  | **Fit indices** | | | | **Class Proportion** | | | | |
| --- | --- | --- | --- | --- | --- | --- | --- | --- | --- |
| **Number of classes** | **BIC** | **SaBIC** | LMR  *p*-value | entropy | **1** | **2** | **3** | **4** | **5** |
| **Linear** |  |  |  |  |  |  |  |  |  |
| 1 | 980.73 | 939.52 |  |  |  |  |  |  |  |
| **2^a^** | **872.76** | **822.05** | **.003** | **.77** | **.76^b^**  **(*n =* 182)** | **.24**  **(*n =* 58)** |  |  |  |
| 3 | 857.34 | 797.11 | .18 | .77 | .61 | .34 | .05 |  |  |
| 4 | 863.84 | 794.11 | .68 | .72 | .04 | .17 | .61 | .19 |  |
| 5 | 868.16 | 788.91 | .10 | .78 | .31 | .03 | .03 | .58 | .06 |
|  |  |  |  |  |  |  |  |  |  |
| **Quadratic** |  |  |  |  |  |  |  |  |  |
| 1 | 985.46 | 941.08 |  |  |  |  |  |  |  |
| 2 | 883.28 | 826.22 | .02 | .76 | .76 | .24 |  |  |  |
| 3 | 866.88 | 797.14 | .17 | .77 | .05 | .33 | .62 |  |  |
| 4 | 877.29 | 794.87 | .23 | .80 | .33 | .04 | .03 | .60 |  |
| 5 | 884.34 | 789.24 | .29 | .68 | .05 | .04 | .31 | .12 | .49 |

^a^ The 2-class linear model was chosen based on the significant Lo-Mendell-Rubin (LMR) test, comparable entropy, Bayesian Information Criterion (BIC) as well as sample-adjusted BIC (SaBIC). Additionally, a comparison of the 2-class linear versus 2-class quadratic models indicated that the quadratic term was not significant in any of the two classes.

^b^ The resilience group.

Table S10. Fit statistics for GMM of caregiver reported peer competence from Wave 1 to Wave 3 with 1- to 5-class solutions and sample posterior probabilities in each latent class.

|  | **Fit indices** | | | | **Class Proportion** | | | | |
| --- | --- | --- | --- | --- | --- | --- | --- | --- | --- |
| **Number of classes** | **BIC** | **SaBIC** | LMR  *p*-value | entropy | **1** | **2** | **3** | **4** | **5** |
| **Linear** |  |  |  |  |  |  |  |  |  |
| 1 | 1205.44 | 1164.23 |  |  |  |  |  |  |  |
| 2 | 942.83 | 892.12 | <.001 | .88 | .27 | .73 |  |  |  |
| 3 | 882.59 | 822.36 | .01 | .83 | .57 | .15 | .28 |  |  |
| 4 | 869.44 | 799.71 | .37 | .82 | .15 | .05 | .28 | .51 |  |
| 5 | 870.15 | 790.91 | .22 | .84 | .05 | .27 | .01 | .52 | .15 |
|  |  |  |  |  |  |  |  |  |  |
| **Quadratic** |  |  |  |  |  |  |  |  |  |
| 1 | 121.90 | 1166.52 |  |  |  |  |  |  |  |
| 2 | 951.55 | 894.50 | .02 | .89 | .73 | .27 |  |  |  |
| **3^a^** | **882.66** | **812.92** | **.04** | **.84** | **.57^b^**  **(*n =* 137)** | **.29**  **(*n =* 69)** | **.14**  **(*n =* 34)** |  |  |
| 4 | 862.81 | 780.40 | .06 | .85 | .28 | .56 | .06 | .10 |  |
| 5 | 866.39 | 771.30 | .23 | .85 | .52 | .07 | .28 | .10 | .03 |

^a^ The 3-class quadratic model was chosen based on significant Lo-Mendell-Rubin (LMR) test, comparable entropy, Bayesian Information Criterion (BIC) as well as sample-adjusted BIC (SaBIC).

^b^ The resilience group.

**Supplementary Material 2 Figures**

Figure S1: Growth mixture model of youth reported intrusive ruminative grief from Wave 1 to Wave 3 for the 3-class linear model.

| Resilience (n = 87, 36.3%) Y =2.149-0.085*X  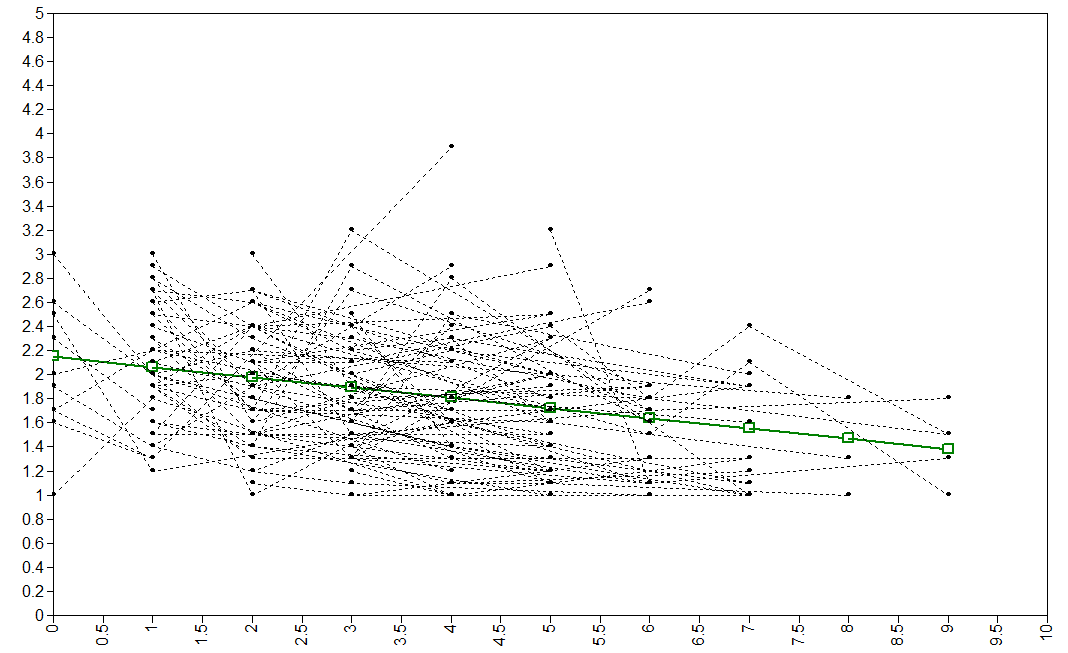 | Line 1 (n = 42, 17.5%) Y =4.17-0.02*X 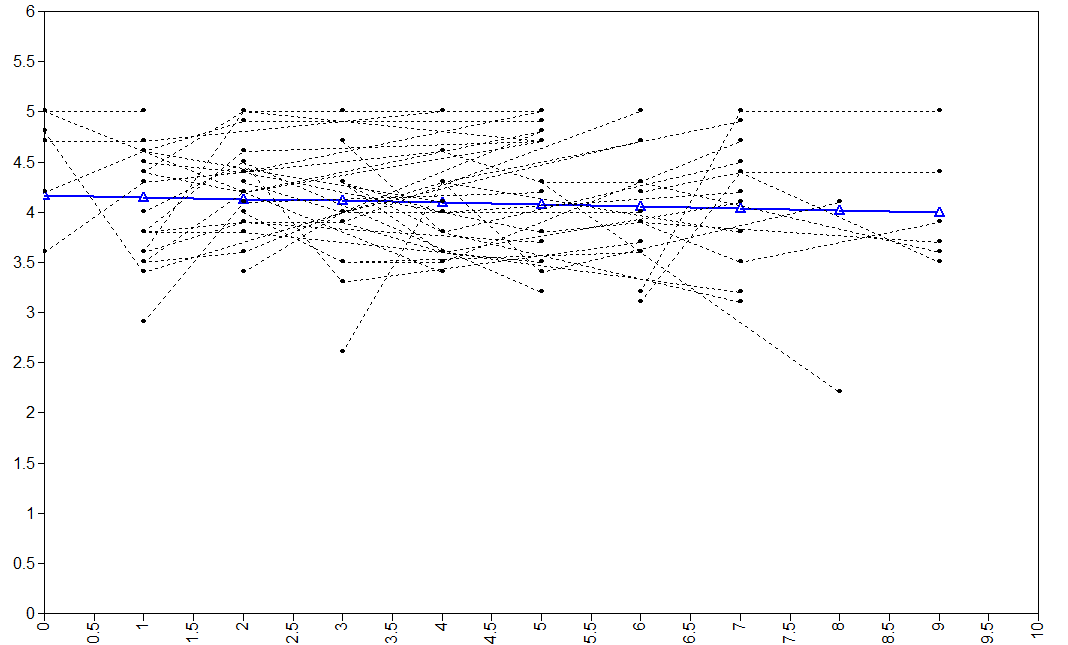 |
| --- | --- |
| Line 2 (n = 111, 45.3%) Y =3.342-0.113*X  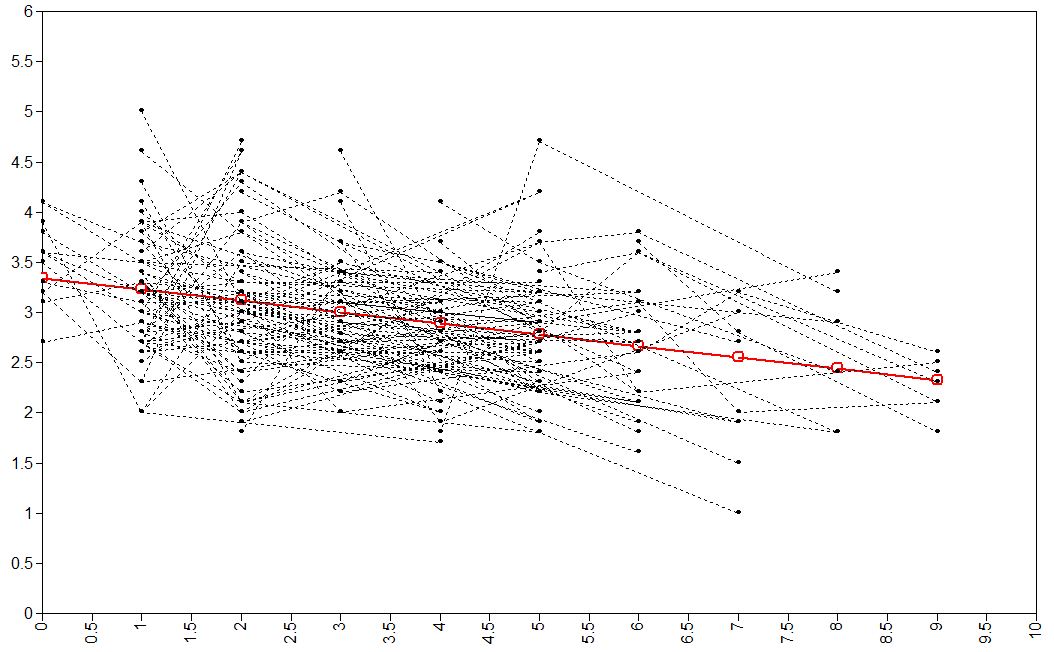 |  |

Figure S2: Growth mixture model of youth reported depression from Wave 1 to Wave 3 for the 3-class quadratic model.

| Resilience (n = 198, 82.5%) Y = 0.239+0.007*X-0.002*X^2  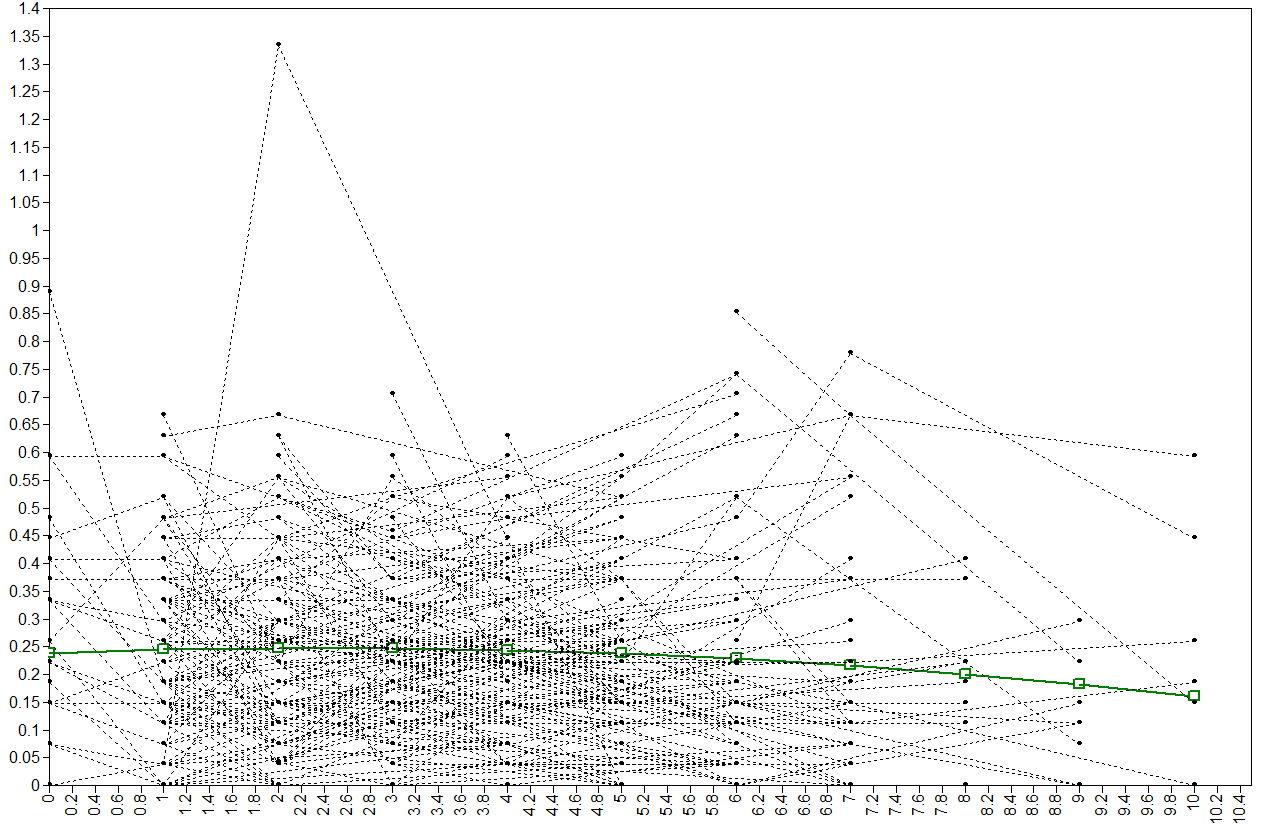 | Line 1 (n = 31, 12.9%) Y = 1.161-0.265*X+0.024*X^2  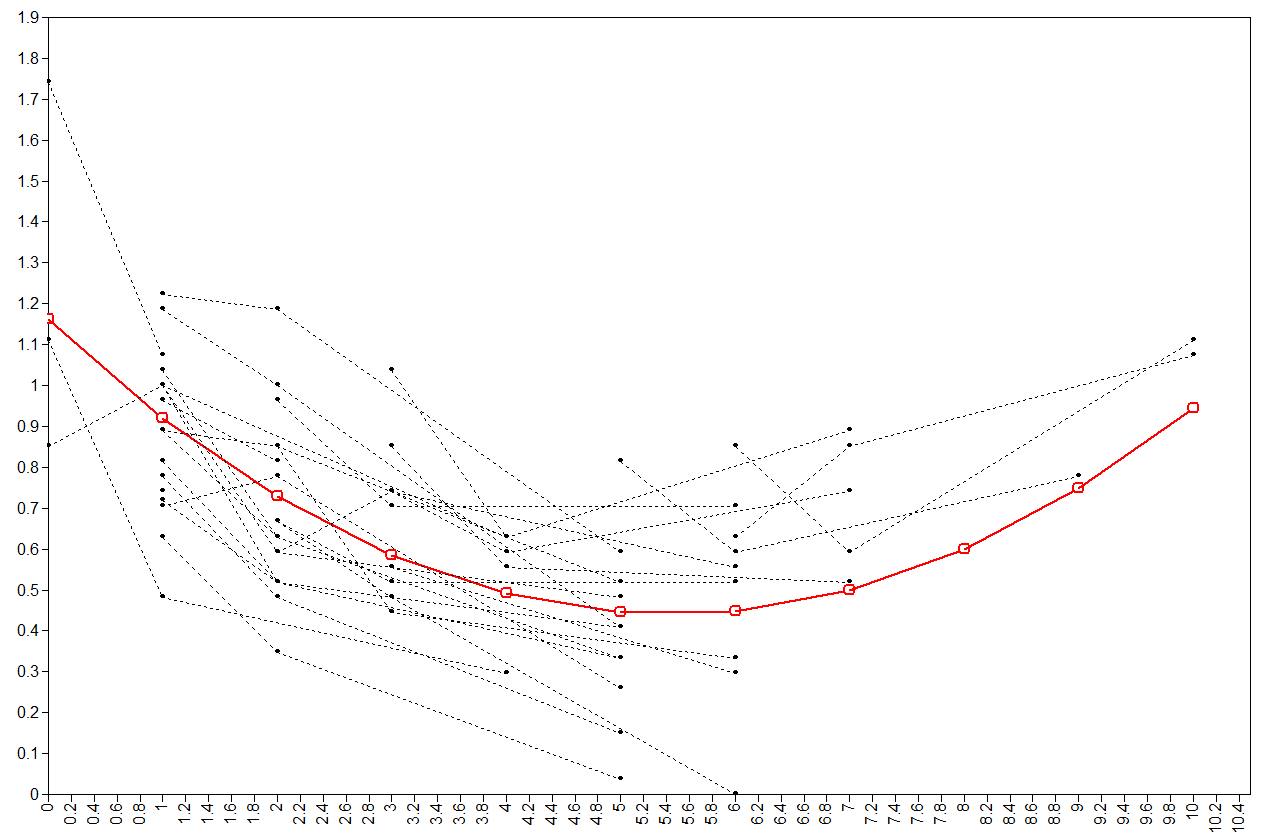 |
| --- | --- |
| Line 2 (n = 11, 4.6%) Y = 0.171+0.331*X-0.041*X^2  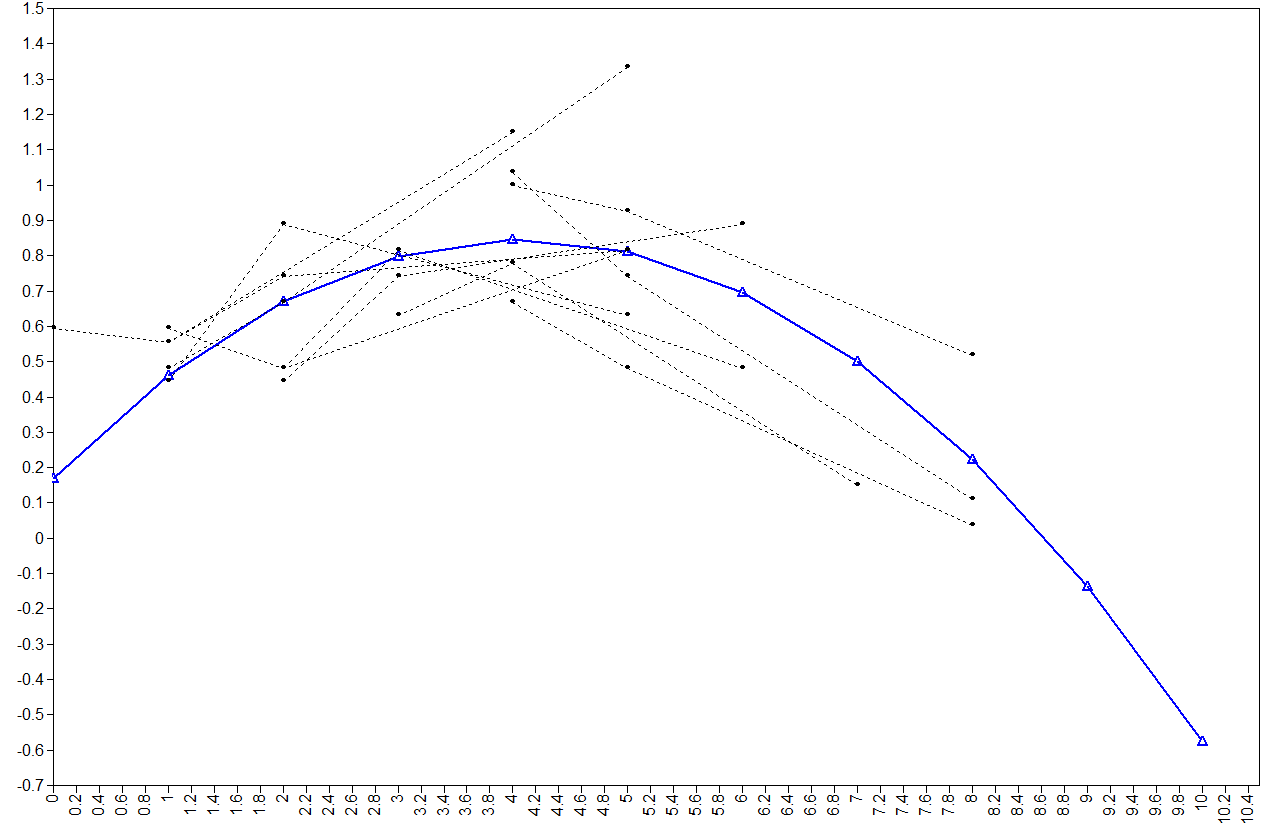 |  |

Figure S3: Growth mixture model of youth reported anxiety from Wave 1 to Wave 3 for the 3-class quadratic model.

| Resilience (n = 154, 64.2%) Y = 1.207-0.014*X  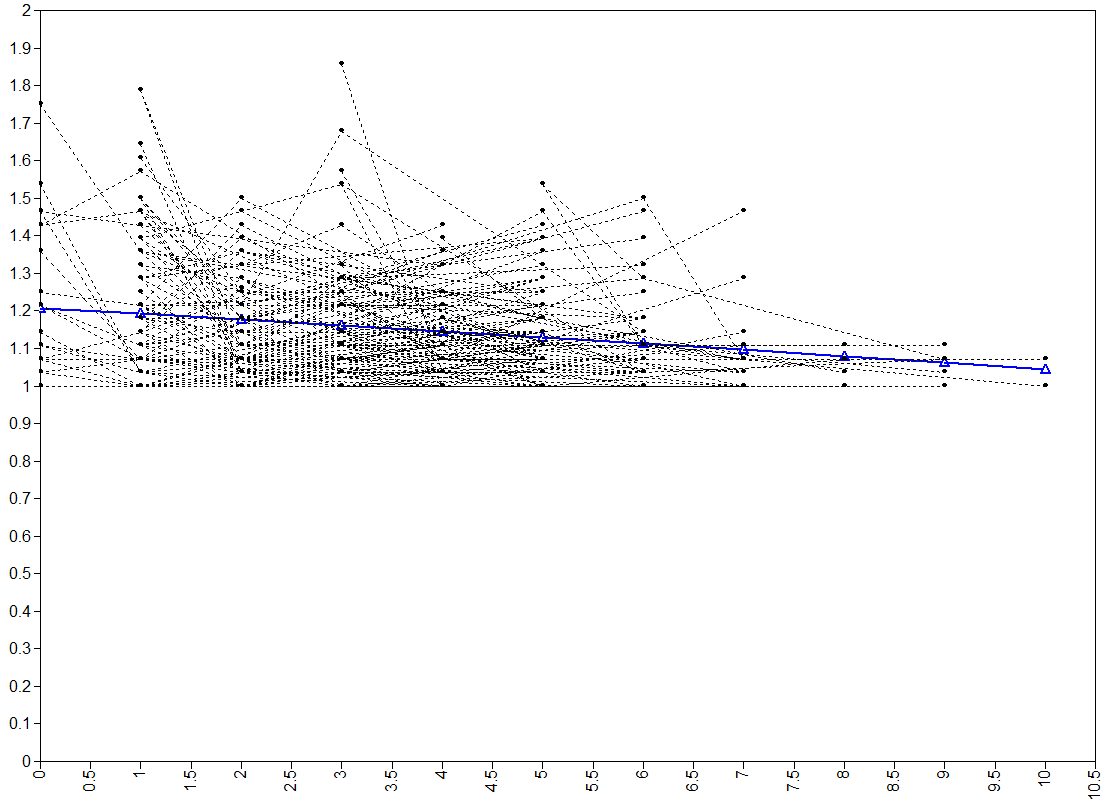 | Line 1 (n = 53, 22.1%) Y = 1.676-0.026*X-0.003*X^2  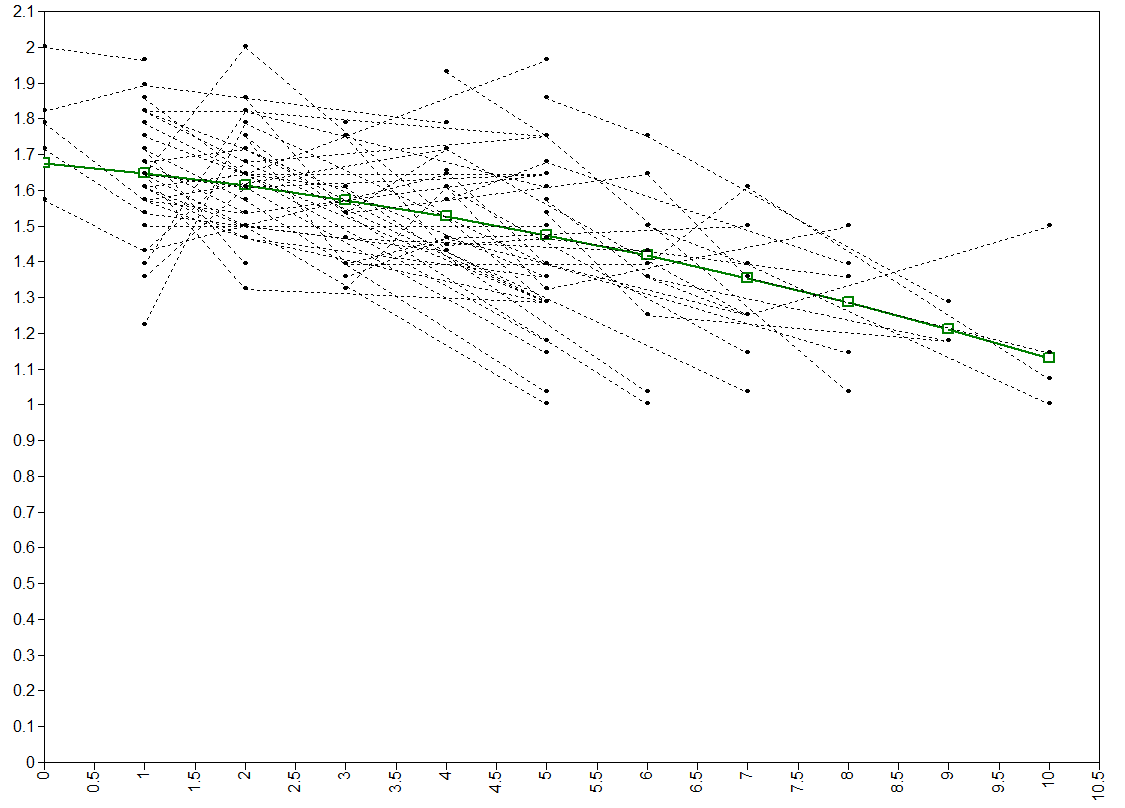 |
| --- | --- |
| Line 2 (n = 33, 13.8%) Y = 1.244+0.103*X-0.006*X^2  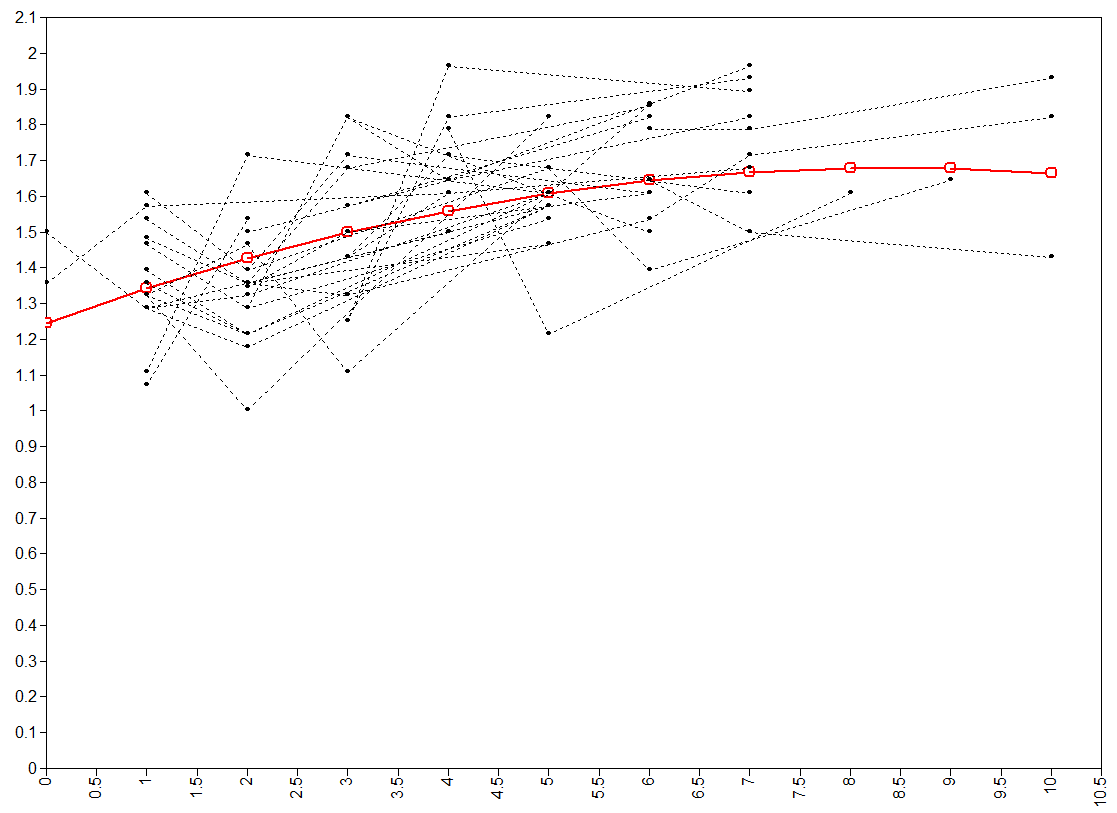 |  |

Figure S4: Growth mixture model of caregiver reported internalizing symptoms from Wave 1 to Wave 3 for the 4-class quadratic model.

| Resilience 1 (n = 47, 19.6%) Y = 45.439-3.92*X+0.403*X^2 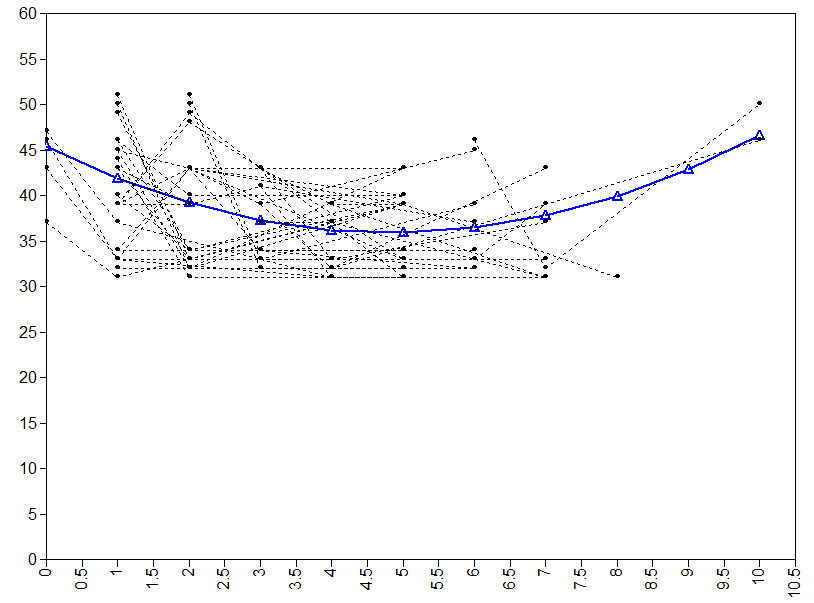 | Resilience 2 (n = 102, 42.6%) Y = 53.60 - 2.358*X+0.121*X^2  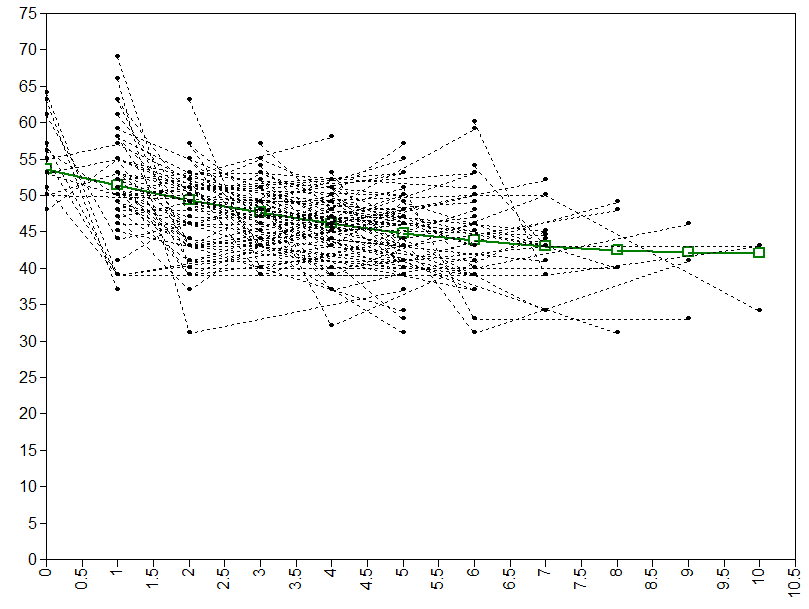 |
| --- | --- |
| Line 2 (n = 77, 32.1%) Y = 63.797- 1.743*X+0.094*X^2  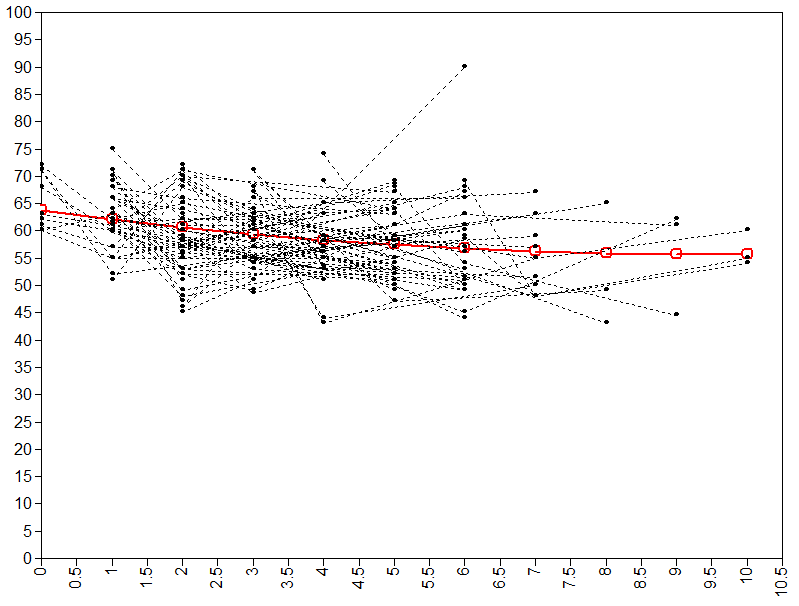 | Line 2 (n = 14, 5.8%), Y = 74.166 -1.462*X+0.083*X^2  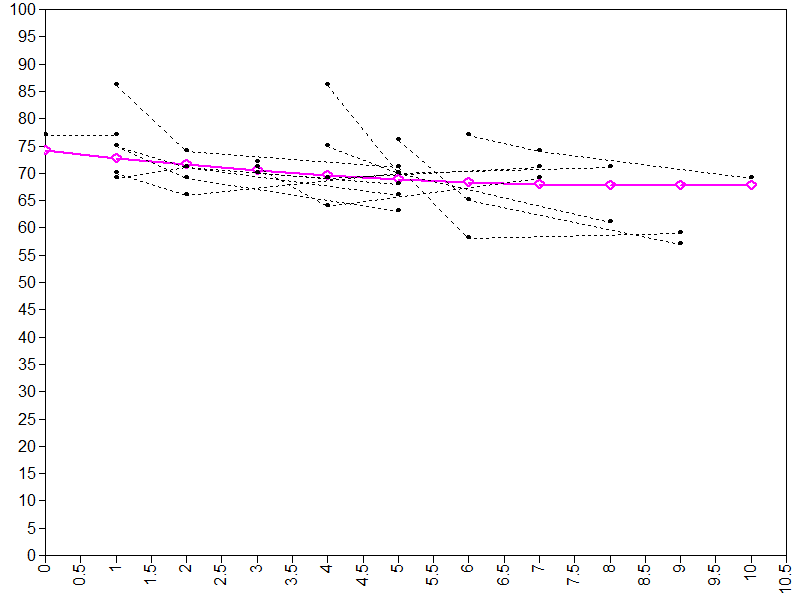 |

Figure S5: Growth mixture model of youth reported externalizing symptoms from Wave 1 to Wave 3 for the 2-class quadratic model, observed and estimated means for each profile.

| Resilience (n = 149, 62.1%) Y = 42.242+0.692*X-0.127*X^2  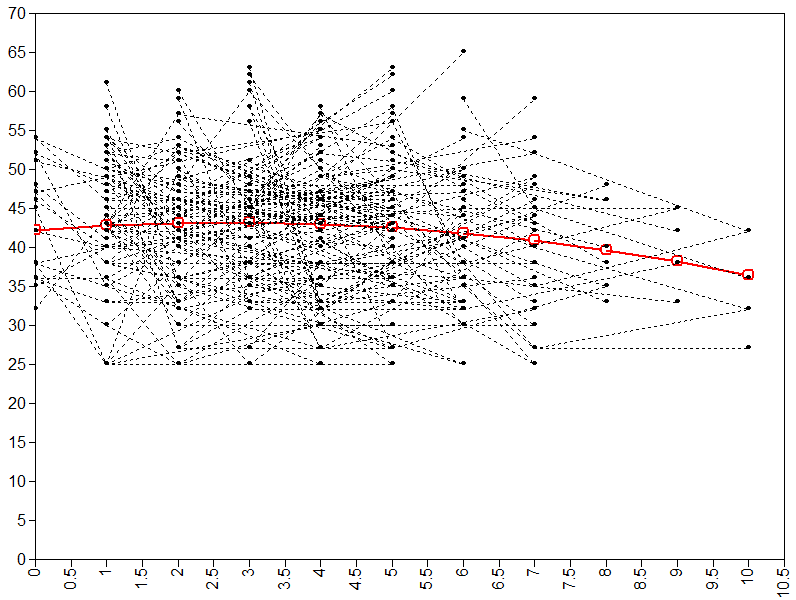 | Line 1 (n = 91, 37.9%) Y = 60.896-2.370*X+0.342*X^2 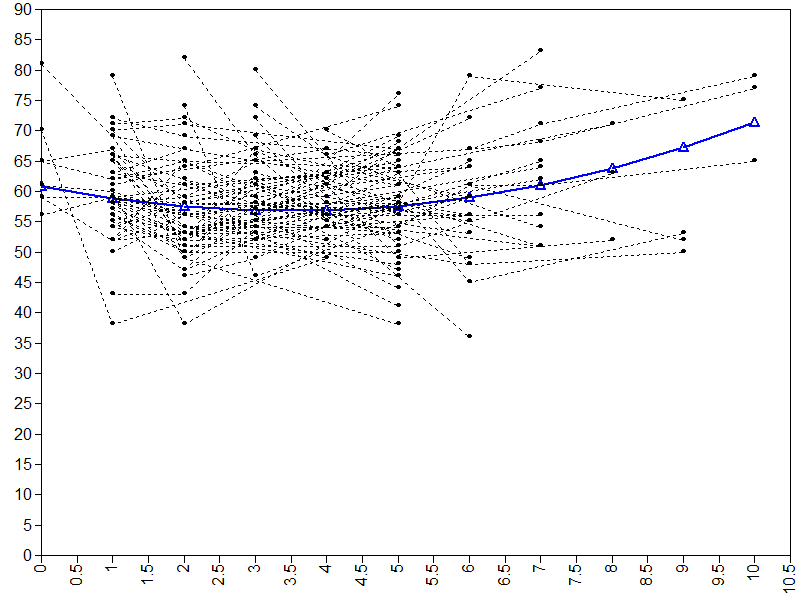 |
| --- | --- |

Figure S6: Growth mixture model of caregiver reported externalizing symptoms from Wave 1 to Wave 3 for the 5-class linear model.

| Resilience 1 (n = 37, 12.9%)  Y= 37.551 - 0.015*X  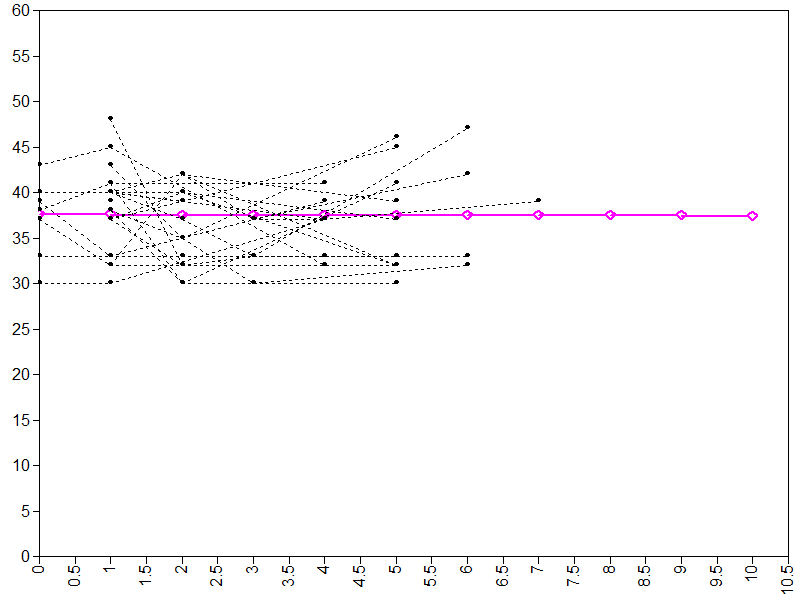 | Resilience 2 (n = 106, 44.2%)  Y= 49.208 - 1.31*X  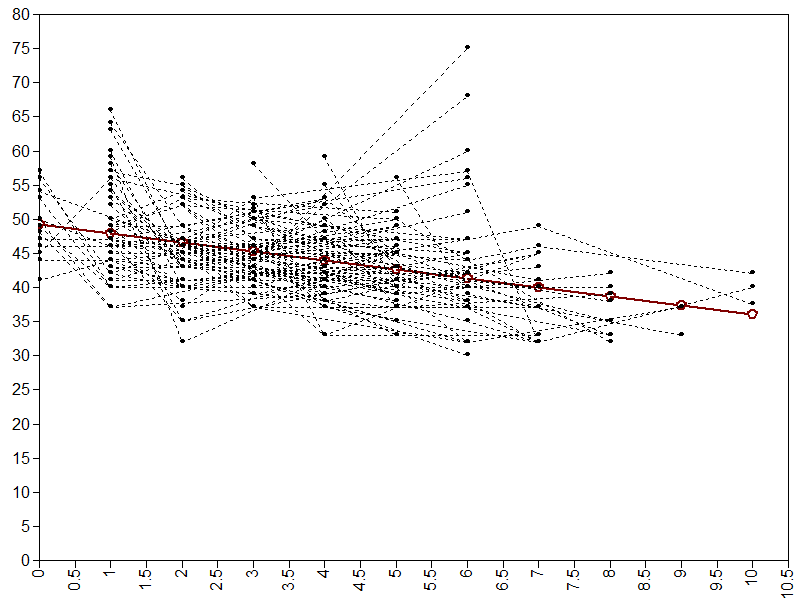 | Line 1 (n = 61, 25.4%)  Y = 60.015 -1.343*wave13  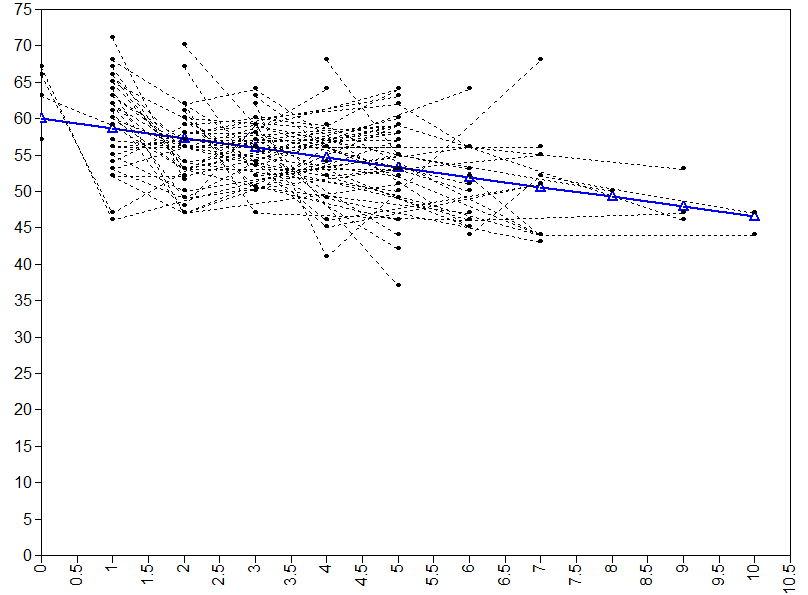 |
| --- | --- | --- |
| Line 3 (n = 31, 15.4%)  Y = 63.058 +0.015*X  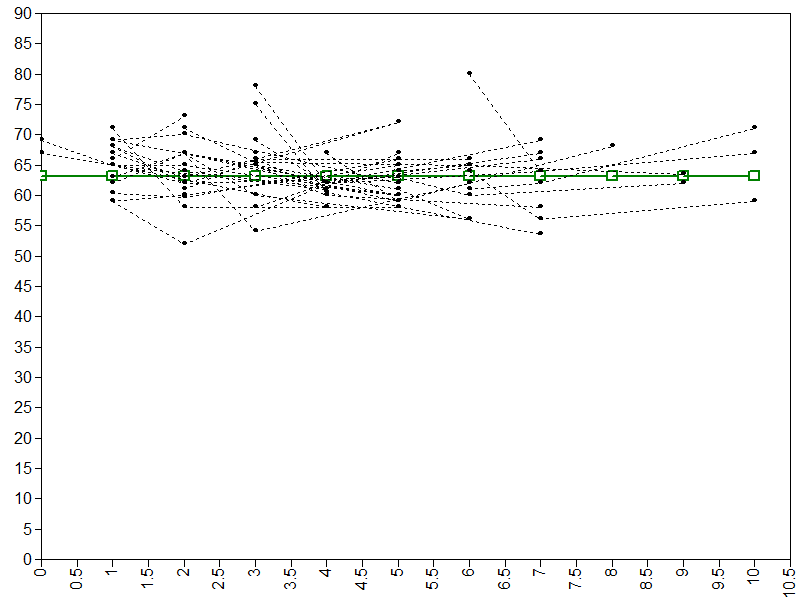 | Line 4 (n = 5, 2.1%)  Y = 76.336 - 0.189*X  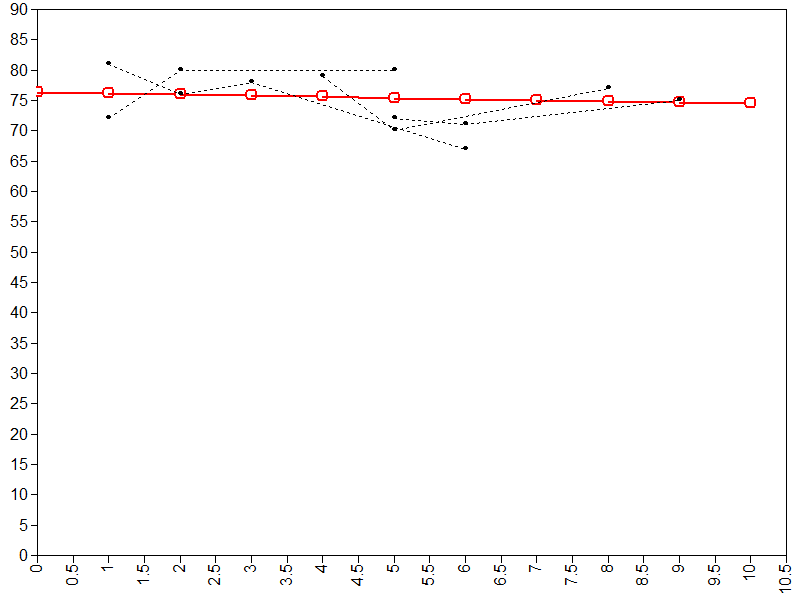 |  |

Figure S7: Growth mixture model of youth reported academic competence from Wave 1 to Wave 3 for the 2-class linear model.

| Resilience (n = 138, 57.5%) Y = 3.436+0.017*X  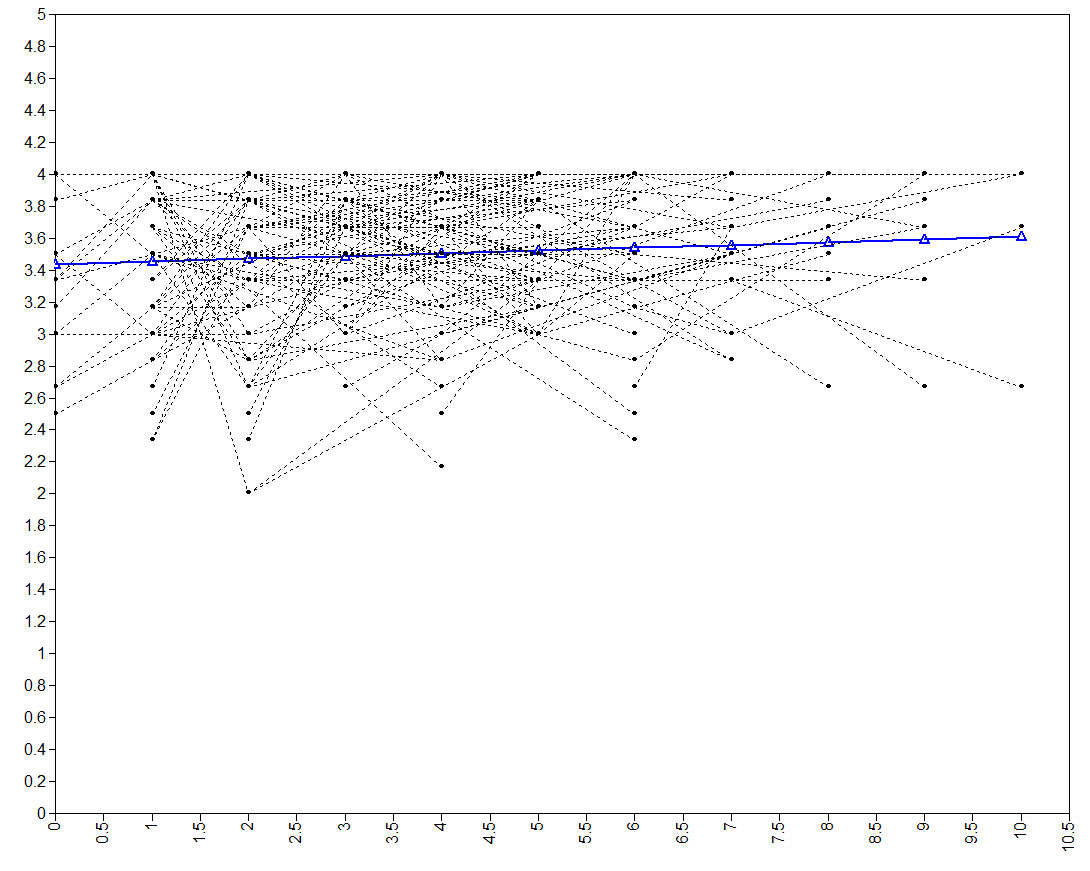 | Line 1 (n = 102, 42.5%) Y = 2.587+0.001*X  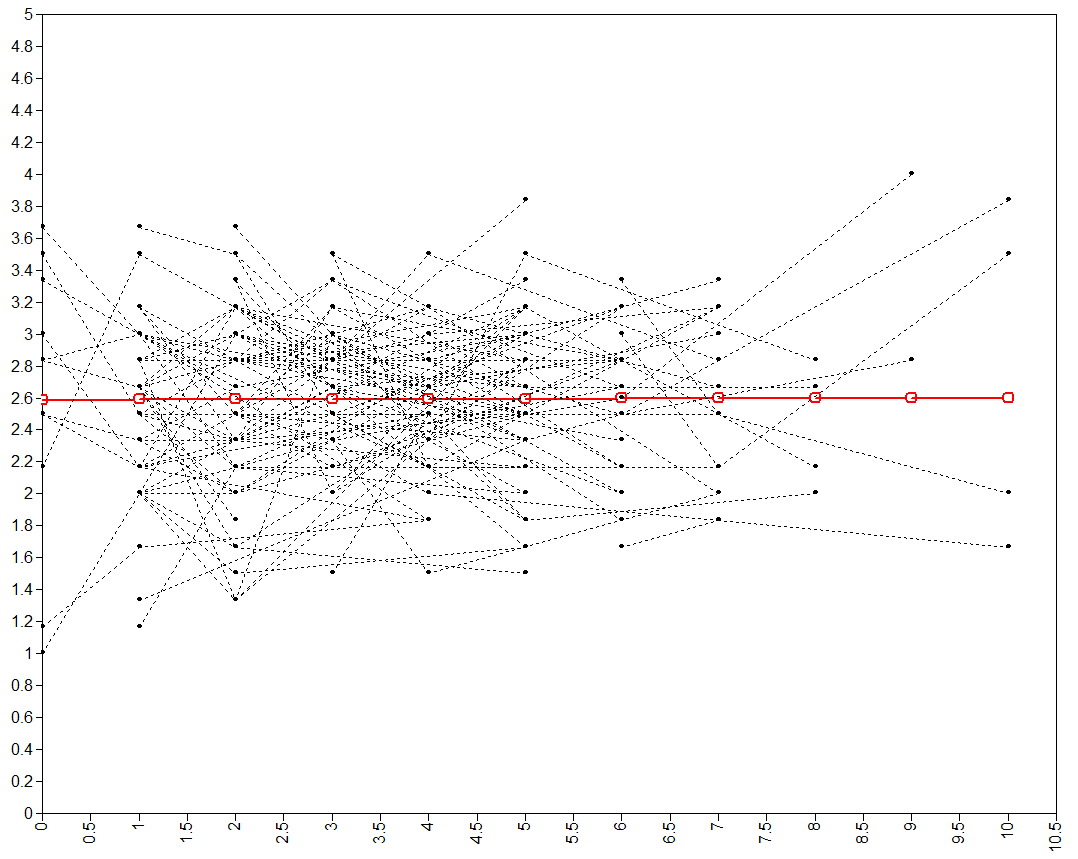 |
| --- | --- |

Figure S8: Growth mixture model of caregiver reported academic competence from Wave 1 to Wave 3 for the 2-class linear model.

| Resilience (n = 164, 68.3%) Y = 3.562+0.006*X  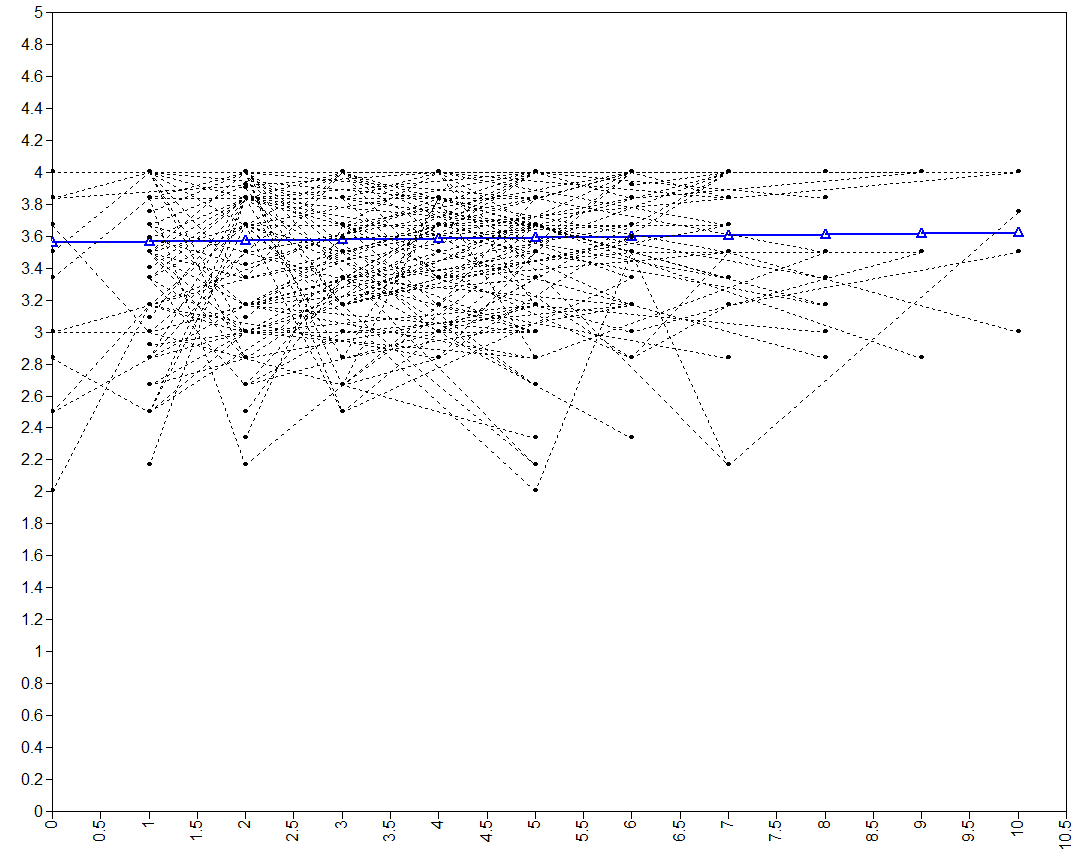 | Line 1 (n = 76, 31.7%) Y = 2.106 + 0.04*X  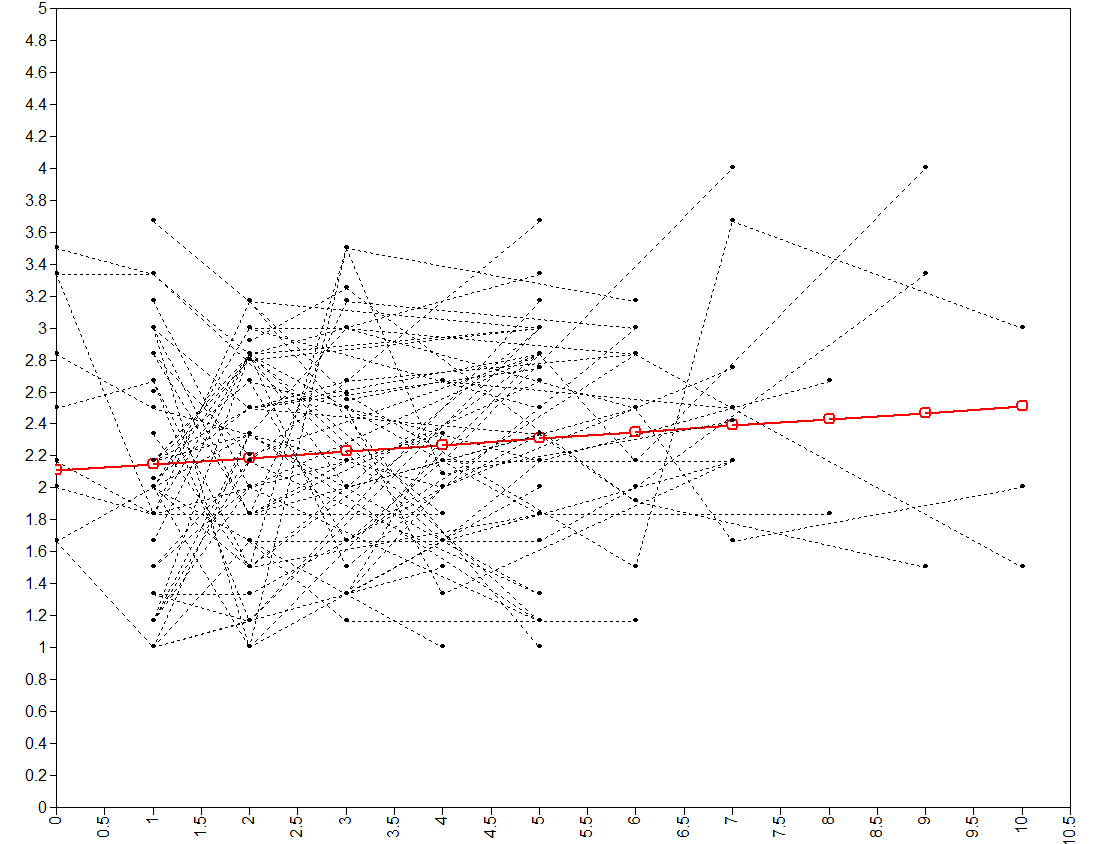 |
| --- | --- |

Figure S9: Growth mixture model of youth reported peer competence from Wave 1 to Wave 3 for the 2-class linear model.

| Resilience (n = 182, 75.8%) Y = 3.535+0.016*X  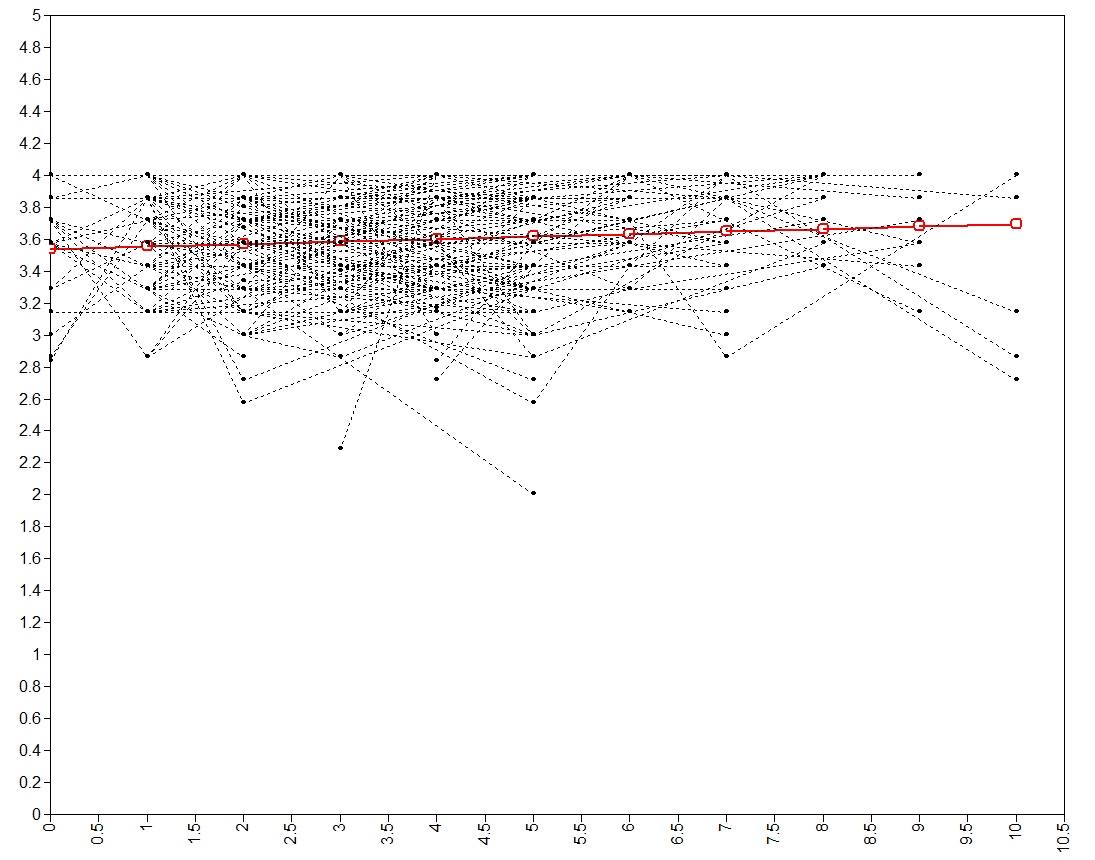 | Line 1 (n = 58, 24.2%) Y = 2.774+0.045*X  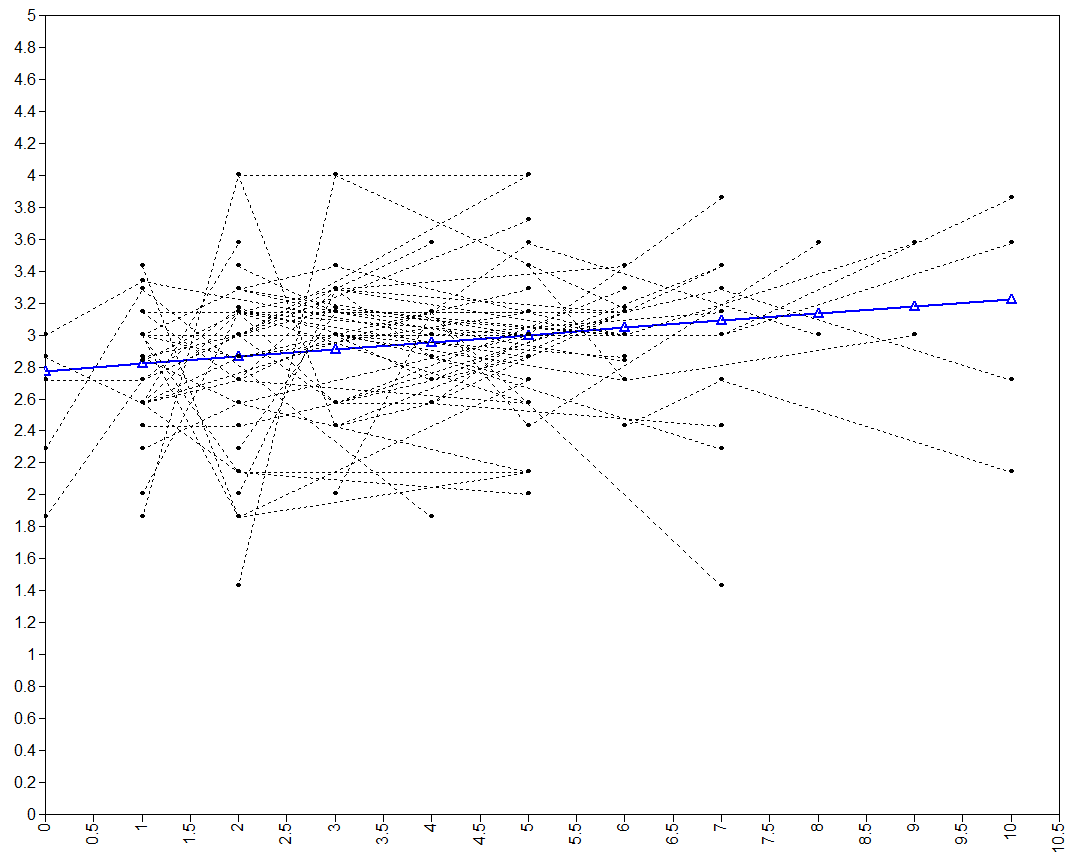 |
| --- | --- |

Figure S10: Growth mixture model of caregiver reported peer competence from Wave 1 to Wave 3 for the 3-class quadratic model.

| Resilience (n = 137, 57.1%) Y = 3.657+0.028*X-0.001*X^2  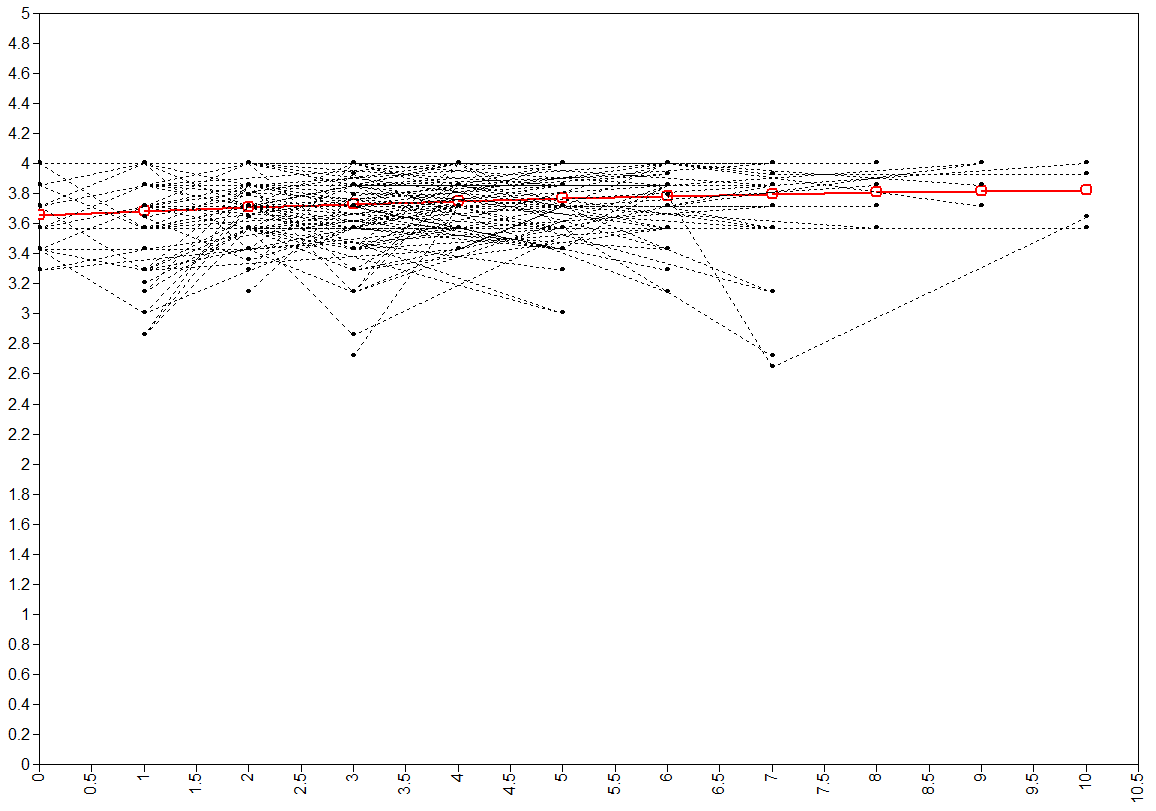 | Line 1 (n = 34, 14.2%) Y = 2.490-0.105*X+0.021*X^2  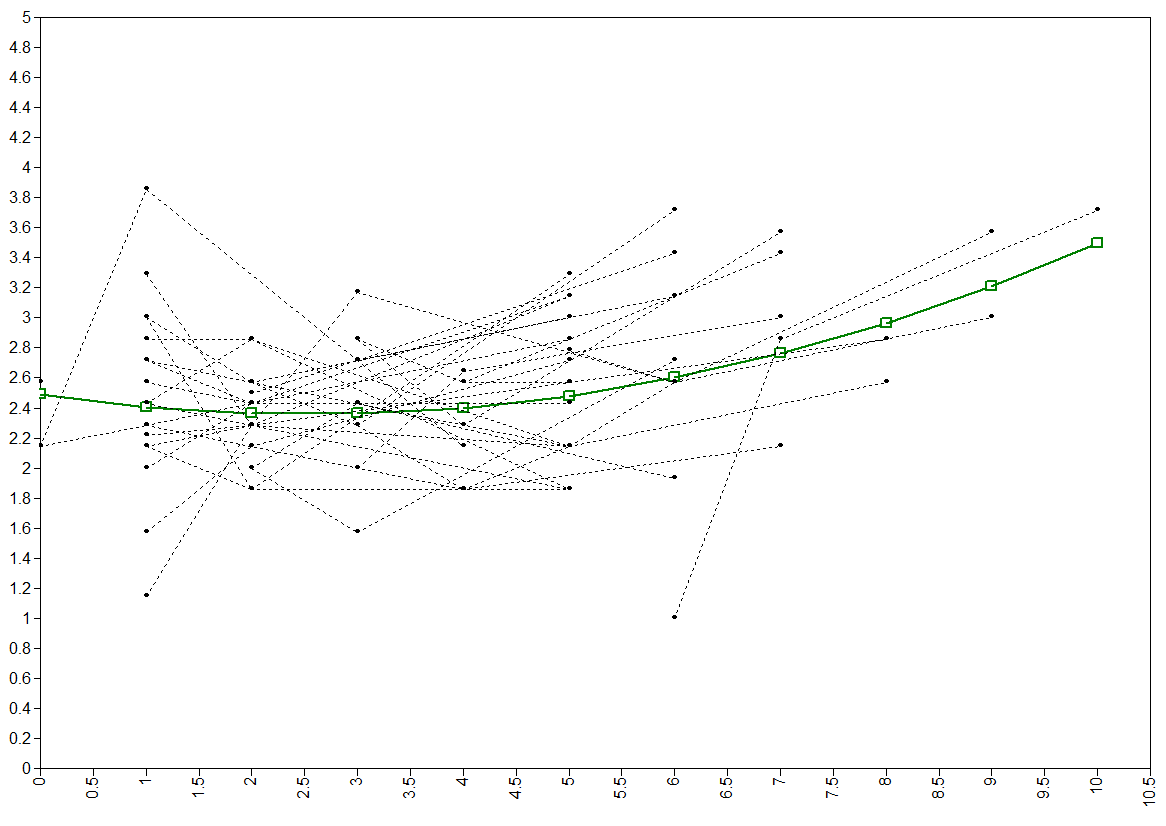 |
| --- | --- |
| Line 2 (n = 69, 28.7%) Y = 2.968+0.106*X-0.011*X^2  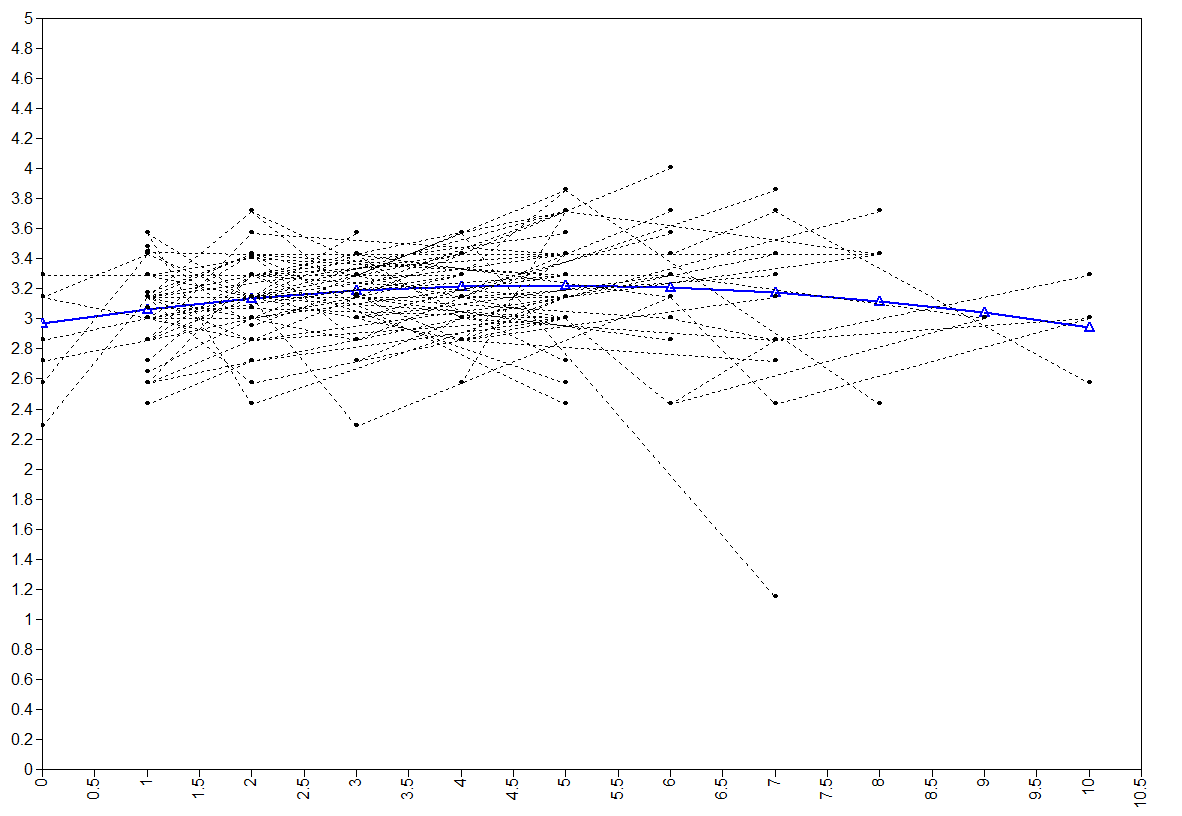 |  |
